# Report on the Progress of Pathology, Practical Medicine, and Therapeutics, during the Years 1842-3-4

**Published:** 1845-07

**Authors:** James Risdon Bennett

**Affiliations:** Assistant Physician and Lecturer on Materia Medica and Therapeutics at St. Thomas's Hospital


PART THIRD.

### Original Reports and Memoirs.

---

### REPORT ON THE PROGRESS OF PATHOLOGY, PRACTICAL MEDICINE, AND THERAPEUTICS,

DURING THE YEARS 1842-3-4.

By James Risdon Bennett, m.d. Edin.
Assistant Physician and Lecturer on Materia Medica and Therapeutics at St. Thomas's Hospital.

This Report is similar in character to those which have already appeared in this Journal. It does not profess to give a complete index to all that has been written on the subject of which it treats, but simply to present a general view of the most important facts and opinions, whether revealing anything new or confirming or refuting what is old. As it is proposed, in a subsequent Report, to furnish an account of the progress of knowledge in the departments of Chemical and Microscopic Pathology, with a few exceptions, no reference has been made to these branches of medical science. Nor has any notice been taken of the important subject of insanity, partly because nothing very new has appeared, with the exception of the results of a more general and extended trial of the non-restraint system of treatment, and partly because any satisfactory notice of the statistical and other reports of the various institutions for the treatment of the insane would have required a larger space than could have been given. And, indeed, the length of period comprised in this Report, from October 1, 1842, to Oct. 1, 1844, has rendered it necessary both to curtail and omit much that might otherwise have demanded notice. The subjects treated of, have been arranged under two divisions. I, Pathology, and II, Practical or Clinical Medicine and Therapeutics. In these and the minor divisions, convenience and perspicuity have been considered rather than strict nosological propriety.

### PATHOLOGY.

### I. General Pathology.

#### 1. Etiology.

*Malaria, its active principle.* Professor Gardner,* of Hampden Sydney College, in an interesting paper on the active principle of Malaria, endeavours to show that sulphuretted hydrogen is the active agent in the production of the fevers of malarious districts, both maritime and inland. His arguments and facts are arranged under the five following propositions: 1st. "Sulphuretted hydrogen gas exists in the stagnant waters and atmospheres of certain marshes." In support of this proposition he adduces the authority of Professor Daniel, who in 1841, found large quantities of the gas in specimens of water sent from several of the African rivers and adjoining seas,—of Mr. Gardner of London, who found it in water from the Bonny aud the Lagos,—and that of Dr. Marcet, who detected it in the Yellow Seas. In order to ascertain whether it exists also in inland malarious districts, Dr. Gardner instituted the following experiments. Having carefully cleaned pieces of silver coin by repeatedly boiling them in solutions of caustic potash and alum, he suspended them by silk thread in three small rivers, the Buffalo, Briery, and Appomatox, in the stagnant water of marshes, in small springs, and in the air over rivers and marshes. In marshes and shallow springs the coins became stained in twenty-two hours. In deep rivers it required sometimes a month, and in the air sometimes longer; but in all the experiments the silver was ultimately stained. The next object was to ascertain the causes of the development of the gas. Four conditions he found necessary: decaying vegetable matter, a rich alluvial soil, saturated with *spring water*, (or water

* American Journal of Medical Sciences, April 1843.

which had percolated the soil,) and the action of the summer heat. Decaying vegetables and alluvial soils contain carbon in excess, and are powerful deoxidising agents. If a sulphate be brought into contact with them, it will be decomposed by the destruction of its acid. Vegetables contain sulphates of lime, soda, potassa, and magnesia. Spring water also usually contains sulphates of lime and magnesia, which Professor Daniel has found are decomposed by decaying leaves. The comparative amount of deleterious gas is determined by the amount of the sulphates, for the requisite changes in which, a certain degree of heat is necessary. Dr. Gardner's 2d *proposition* is, "The character of malarious regions is similar to that of those in which sulphuretted hydrogen is generated." In support of this he adduces the facts recorded by Messrs. Laird and Oldfield in their account of the Niger Expedition and numerous others of the same kind, many of which are very striking and decisive. He, however, admits that "it would be premature to state that in every case where bilious fever has been detected, sulphuretted hydrogen also existed. The catalogue of endemics attributed to this cause includes a host of ailments, from ague to yellow fever, typhus, and plague itself. There is some mistake here, either the exciting cause varies, or the whole of these diseases are not produced by miasma. Some of these complaints are undoubtedly produced by other causes." Again, he says, "in some of the cases adduced in the enumeration of places remarkable for malaria, it is questionable whether the means for generating sulphuretted hydrogen exist." "This is the case in all inland positions where it is uncertain that sulphates are found in the waters of the place." 3d *proposition.* "Certain agents have been supposed to give activity to the exhalations arising from marshes, called malaria." Dew being acknowledged to be the vehicle which conveys it, watery vapour has been regarded as the noxious matter. M. Boussingault has recently advocated the theory that *carburetted* hydrogen is the active agent. He detected carbon in the dew of marshes, in the department of Ain, and having ascertained that hydrogen existed in the same situations, he concluded that carbon existed as carburetted hydrogen. This gas is undoubtedly produced wherever vegetable matters are undergoing putrefaction; but, remarks Dr. Gardner, "the conditions which increase the unhealthiness of particular localities do not contribute to the increase of this gas. The most dangerous sites are on the sea-coast, and where sea water finds access to marshes. Those circumstances which augment and even produce malaria (as in the Ligurian marshes and those of South Carolina) are in no way concerned in the development of carburetted hydrogen gas." Under the head of his 4th *proposition*, that "The properties of malaria are fully recognized by the profession," he refers to the principal of the best established facts in reference to the outbreak, spreading, arresting, and extinction of malarious diseases: and then gives as his 5th *proposition*, "Sulphuretted hydrogen is the active agent in the production of those forms of malarious fevers met with on the sea-coast, and the diseases belonging to the same class found inland." The sulphur is supposed to exist in malaria as a component of an organic body, containing carbon, hydrogen, sulphur, and water. In a supplementary letter, Dr. Gardner attempts to account for the absence of malaria from certain marshes—viz., those around Boston, (U. S.), and the bogs of Ireland—by supposing that iron, or zinc, or other metals exist in the subsoil, and that these by uniting with the sulphur prevent the development of sulphuretted hydrogen. These views of Dr. Gardner are in opposition to the opinion expressed by Dr. Pritchett,* in his account of the African remittent fever, for he denies the existence of sulphuretted hydrogen in the water of the Niger, and maintains that, "even if it were evolved from the waters of the west coast of Africa, it would not explain the fevers which there prevail. He indeed denies altogether the miasmatic origin of the fever, which he attributes to the ordinary atmospheric influences of hot climates.

* Some Account of the African Remittent Fever, which occurred on board Her Majesty's steamship Wilberforce, &c.; London, 1843.

Dr. M'William also* concurs in denying the existence of sulphuretted hydrogen in the waters of the Niger, and in maintaining that what was detected in the specimens previously sent to England originated in the decomposition of the contents of the bottles. Dr. Minzi,† of the Central Hospital of Terracina, with a view to determine whether in the genesis of paludial fevers there was really any special miasmatic principle, collected, by means of an apparatus containing a frigorific mixture, the dew which fell in the vicinities of Rome and Terracina. Of this he and several other persons drunk portions varying from ℥ij to ℥vj without any ill consequence. Wounds on the legs of two peasants were also washed with the dew water without any bad effect. He concludes therefore that the miasmatic principle, if any such exist, does not reside in the dew of malarious districts.

*Geological causes of fever.* Dr. Heyne, of Madras,‡ in an important paper on the hill fevers of India, ascribes as their principal cause the geological character of the hills among which they occur. Hill fever, he says, invariably exists among certain descriptions of hills, whilst others of a different geological character are as invariably free. Wherever the iron granite, or magnetic ironstone rocks occur, there will be fever, whilst the hills whose strata are free from ferruginous compounds are equally free from the destructive fever to which Dr. Heyne alludes. The ferruginous granite rocks are remarkable for their disintegration, not only separating in the hot season into large masses of many tons, but crumbling also into their constitutent parts, and forming an abundance of sand which is attracted by the magnet, though this is not affected by the rocks in masses. Dr. Heyne therefore attributes the fevers of these hills to the magnetic or electric fluid which seems to exist in the greatest abundance in the iron hornblende, and is disengaged in great quantity in the hot season. The first rain that cools the atmosphere to 74° puts a stop to the discharge of the magnetic or electric principle, and to the further progress of the fever. Epidemic fevers in Madras, he states, are preceded by electrical phenomena.

*Contagion.* Some very valuable facts and observations on the importation and propagation of plague and other contagious diseases are contained in the extracts from an unpublished work by Dr. Furguson, Inspector-General of Army Hospitals, which have appeared in the 'Edinburgh Medical and Surgical Journal' for January and July, 1843.

*Hereditary transmission of intermittent fever.* Dr. Brunzlow,§ of Magdeburg, relates the following as an instance of the hereditary transmission of ague. A woman, aged 34, was seized in the second month of pregnancy with tertian ague, which after several weeks was cured by bark. It returned with the quartan type, and lasted to the seventh month, returned a second time in the eighth month, and was not finally cured till in the course of the ninth month. She gave birth to a thin, feeble child, which when some months old, she observed, was still thin and weakly, cried, shook, and had much heat every fourth night. It was cured by frictions of quinine and lard to the epigastrium and in the axillæ and the internal use of quinine. Three attacks only occurred after this treatment was commenced, and the child afterwards became robust.

*Measles, transmission by inoculation.* Dr. M. Von Katona,‖ in a very malignant and wide-spread epidemic of measles in the winter of 1841, inoculated 1122 persons, with a drop of fluid from a vesicle, or with a drop of the tears of a patient with measles. It failed in seven per cent. of those on whom it was tried; but in all the rest the disease was produced in a very mild form, and not one of them died. At first a red areola formed round the puncture, but this soon disappeared; on the 7th day fever set in, with the usual prodomi of measles; on the 9th or 10th, the eruption appeared; on the 14th, desquamation commenced, with decrease of the fever and the eruption; and by the 17th, the patients were almost always perfectly well.

* Medical History of the Expedition to the Niger, &c.; London, 1843.

† Bulletino delle Scienze Mediche, Nov. and Dec. 1843, p. 388.

‡ Provincial Medical and Surgical Journal, Sept. 3, 1842; from Madras Medical Journal.

§ Bullet. gén. de Thérap., 15 and 30 Nov. 1842, p. 380. ‖ Oesterr. Med. Wochensch. July 16, 1842.

*Ætiology of diseases of the heart.* Dr. Flögel* thinks that among the determining causes of cardiac disease, immoderate, long continued, or even only momentary bodily efforts, especially of the muscles of respiration, of such kinds as interfere with the free performance of respiration, have not received the attention their importance deserves. He gives five cases in which the patients referred their cardiac symptoms to muscular efforts, and insists on the importance of these facts in reference to prophylaxis.

*Hemorrhage, meteorology of.* In a paper having this title, Dr. Joslin† proposes to examine among the various causes whose combined influence determines the time when a spontaneous hemorrhage shall occur, whether the condition of the atmosphere has an influence so great as to be discoverable by a careful comparison of medical and meteorological observations. The examination was restricted to cases of hæmoptysis and uterine hemorrhage, occurring in his own practice, in three consecutive years, and those cases only were selected in which the exact hour and day of attack were known.

1. Season and temperature. The greater number of cases occurred in June and September, hæmoptysis taking the lead in the former, and uterinehemorrhage in the latter month. Neither the extreme ofheat nor of cold appeared to be among the most influential causes. But by examining the dew point and the difference between it and the temperature, some depression of temperature seemed to be a usual concomitant of hemorrhage. The average depression of the thermometer below the monthly mean was 38, but the fall was greater for hæmoptysis.

2. Hygrometric condition. There did not appear to be any relation between the hygrometric condition and the occurrence of hemorrhage, except in so far as that change of temperature (which is attended by a corresponding diminution of vapour) entails an alteration of hygrometric state.

3. Barometrical condition. The barometric results were more remarkable than the hygrometric or thermometric, and in many respects opposed to generally received opinions. During the 24 hours preceding the attack, the instances in which the barometer was rising were nearly equal to those in which it was falling. This correspondence applied to both sets of cases. Before the uterine hemorrhage the barometer rose 13 times, and fell 14, and the same occurred before hæmoptysis. There was, therefore, a slight tendency to depression, but not such as to justify any general conclusion. The case was different for the days of attack: on these the barometer was generally falling, and in a greater proportion of cases than could be with any probability attributed to accident. Out of 54 cases, it was in 34 falling, at the time of attack, in 18 rising, and in 1 stationary. The proportion of cases in which the barometer fell was almost exactly the same for both classes of hemorrhages; being 17 to 9 for the uterine, and 18 to 9 for the pulmonary. But that this influence of diminished atmospheric pressure was not mechanical, seems proved by the fact that the effect was not at a maximum when the pressure was at a minimum, and the blood-vessels thus in an unusual degree deprived of support; for the barometer, though generally falling, was not *low*, but on an average about one third of one tenth of an inch *above* the *mean* height for the year. The conclusion from all the barometrical facts was, that at the commencement of the attack of hæmoptysis, or uterine hemorrhage, the barometer is *generally falling*, and from some points *above* the *mean*.

4. Storms. The observations made on the relations of storms of rain or snow to the occurrence of hemorrhage tend to the conclusion that the atmospheric condition *preceding a storm* is more conducive to hemorrhage than that which succeeds one. This conclusion is confirmed by comparing the three days which precede, with the three which immediately succeed. The proportion of the former

*Oesterreich. Med. Wochenschr. July 15, 1843. Zur Aetiologie der Herzkrankheiten von Dr. Jos. Flögel.

† Amer. Journal of Medical Sciences, "On the Meteorology of Hemorrhage," by B. F. Joslin, M.D.; New York.

which were stormy, was for both kinds of hemorrhage collectively only, thirty-six and a half per cent., that of the latter fifty-one and a half.

On reviewing all the meteorological circumstances, the mean results, whether barometrical, thermometrical, or hygrometrical, all conspire to point to a time of transition from a fair and *dry* to a more foul and *stormy* period, or at least to a time characterized by great electrical changes, and especially to the development of much free electricity in the upper regions of the atmosphere, by the precipitation, and even crystallization of aqueous vapour.

*Cretinism, causes of.* Dr. Roesch* having been ordered by the government of Würtemburg to inquire into the causes of cretinism, examined 3000 cretins in different localities where the disease was endemic. The following are the more important of the conclusions at which he arrived. 1. Cretinism is sometimes sporadic, but in certain localities is endemic. 2. It is hereditary, with the ordinary laws and exceptions to which hereditary diseases are subject. 3. The conditions for its development are hereditary predisposition, and the action of certain influences on the parents (such as want, deficient food, unwholesome habitations, excessive labour, and debauchery,) and accidental causes acting on the child, during the period of its physical and intellectual development. 4. These accidental causes are certain atmospheric and geological conditions peculiar to certain localities. Impregnation of the water with gypsum, or lime, or melted snow, appeared to Dr. Roesch to exert no evident influence, for he met with cretins, where the water was quite pure. But humidity of the air, he thought, played an important part. Cretinism is never endemic in plains, or on elevated "plateaux," whilst it is found in valleys and "bas fonds" abounding in moisture. It does not exist in cold countries where sudden variations of temperature are rare. All the localities in which it is endemic agree in being humid, foggy, and exposed to sudden changes of temperature, often very hot in the middle of the day, and cool or even cold in the morning and evening. Goitre constantly accompanies cretinism, is indicative of it, and is developed under the same conditions. These views of Dr. Roesch correspond exactly with those of M. Marchand, published in his inaugural thesis of 1842.†

*Defective expansion of the lungs as a cause of disease.* In the Gulstonian Lectures for 1844,‡ Dr. Barlow, in a very philosophic spirit, elucidates some of the consequences ensuing from defective expansion of the lungs in early youth, and refers more particularly to four classes of cases in which pulmonary obstruction is associated with hypertrophy and dilatation of the right heart, pointing out the effects on the liver and the venous circulation generally, and the subsequent occurrence of anasarca. In the first class of cases to which reference is made, the obstruction to the circulation, in the right side of the heart, is produced simply by defective expansion of the lungs and air-passages, at the period of life when the thoracic organs undergo that development which alters their previously existing relation to the abdominal organs.

He gives, in illustration, the case of a girl, who, when aged 12, suffered from dyspnea, palpitation, enlarged liver, ascites, and anasarca, which, after being relieved from time to time, ultimately proved fatal at the age of 15. The chest was narrow and ill developed, the mammæ and genitals were infantile, the liver much enlarged and myristicated—enormous dilatation of the right auricle and ventricle with some hypertrophy—pulmonary artery *small*—valves healthy—right auriculo-ventricular opening enlarged. The left auricle and ventricle were dilated, but much less so—lungs compressed and exsanguine, but structurally healthy—trachea small, and bronchi compressed. All these consequences are referred simply to defective expansion of the lungs, and consequent increased action of the right heart to overcome the impediment to the discharge of its contents.

* Gazette Med. de Strasbourg, Nov. 1842; and Bullet. Génér. de Thérapeutique, 15 et 30 Dec. 1842.

† See also Beobachtungen u. Bemerkungen über den in Oesterreisch haüfig vorkom. Cretinismus. Von Dr. Shausberger, in Oesterreisch. Med. Wochens. 29 Oct. 1842.

‡ Medical Gazette, vol. i, 1843-4, pp. 705-53-85.

In a second class of cases similar results ensue from defective expansion of the lungs, arising from mechanical pressure exerted on them or the air-passages, e.g. in deformity of the spine or chest, or pleurisy and consequent adhesions; unless the opposite lung take on a compensating action, when, in consequence of its increased activity, there is great danger of tubercular deposition. His third class includes a set of cases the true nature of which he thinks has hitherto escaped the notice of pathologists, in which the obstruction to the circulation on the right side of the heart is the result of *pericarditis acting mediately through the impediments which it offers to the respiratory movements.* The state of respiration so characteristic of pericarditis cannot, he argues, continue long in a person whose growth is not yet completed, without offering great obstruction to the development of the lungs, and thus inducing important changes in the right side of the heart, and the evils thence resulting. The consequences of pericarditis are thus very different in the adult, from those ensuing in a person whose frame is not yet fully developed. Dr. Barlow denies that hypertrophy of the heart is a necessary consequence of pericardial adhesion, and adduces a remarkable case illustrative of this, in which a complete ring of ossific matter (deposited in the false membrane, forming the medium of adhesion between the two surfaces of the pericardium), surrounded the base of the heart. The man had been the subject of rheumatic pericarditis two years before, The heart itself was not larger than natural. The true cause of hypertrophy and dilatation, with pericardial adhesions, is the impediment to the circulation through the lungs, and not impeded action of the heart from its being shackled by the pericardial adhesions. In connexion with this subject, he also alludes to the arrest of the heart's growth and inability to carry on the circulation from this cause, as an occasional consequence of pericarditis in young people, and cites a remarkable case of atrophy of the heart.

In the fourth set of cases the defective expansion of the lungs is the consequence of obstruction in the left heart with narrowing of the mitral orifice.

*Contagious cells, Inoculation by means of.* Dr. Klencke* in the 'Arch. für die gesammte Med.' states that he has succeeded in communicating to healthy animals—carcinoma, tubercle, melanosis, condylomata, warts, ozœna, and coryza—charbon (malignant pustule), and hydrophobia—by inoculating with the cells of these several diseases, and mentions as important practical facts, that the cells of recent coryza are readily destroyed by the action of chloride of lime, but if the disease becomes chronic, the cells disappear and are replaced by the confervæ of ozœna. The cells of charbon are so contagious that it is dangerous to inoculate with them; after having subjected them to boiling water, and kept them in lime for fifteen days, he was able to inoculate a small goat. The cells were obtained from a yellow fluid that trickled from the pustule. He has met with the hydrophobic cells in the excised cicatrix of a bite which had given reason to fear hydrophobia, and in the foam from the mucous membrane of the cheek and the salivary glands. These cells are dissolved or rendered inert by boiling water, the mineral acids and chlorine water.

The lymph of variola, and the acute exanthemata is inoculable in proportion as it abounds in contagious cells.

[These statements are corroborated by some facts recorded by previous writers, Gooch, Mayo, and Langenbeck.]

*Phthisis, Influence of employment on.* From an elaborate and valuable paper by Dr. Guy† on the influence of employment in the production of phthisis, the most important conclusions to be drawn are,—that the ratio of cases of pulmonary phthisis to those of all other diseases is highest, both in the male and female sex, among those following in-door employments, and in the case of men, varies inversely with the amount of exertion, being highest where there is least exertion. Neither a constrained posture, nor exposure to a high temperature, nor a moist atmosphere, appears to have any marked influence in inducing con-

* Prov. Med. and Surg. Journal, No. 156.

† Journal of the Statistical Society.

sumption. The ratio of pulmonary phthisis to all other diseases is highest among men exposed to the inhalation of dust, and high among the intemperate. The age at which the disease occurs is early in proportion as the occupation is such as to present a high ratio of cases. The practical inference deducible from these observations is, that the predisposed to phthisis should choose out-door occupations, and among in-door employments, those entailing most exercise, and that they of all others should avoid intemperance and the inhalation of dust. Dr. Jackson,* however, in his analysis of 604 dissections of persons dying of all diseases, in the course of ten years, in Boston, (U. S.) says that intemperance certainly does not appear to develop phthisis, and that of 35 drunkards, 26 presented no trace of tubercle.

*Calculous diseases.* Dr. J. Jackson, of Calcutta,† in a letter to Mr. Crosse, of Norwich, states that the assertion that calculous diseases do not exist in tropical climates is far from the truth, for that in the midland provinces and in upper India there are few districts where these diseases are not prevalent among the lower classes, who are badly fed, and live on an unleavened bread, similar to the Norfolk dumpling. Few cases, however, are met with among the aged.

*Scrofula.* M. Lugol's treatise on the Causes of Scrofula‡ can scarcely be said to be correctly designated, inasmuch as he appears to consider hereditary predisposition as the *sole* cause, all others merely influencing its development, form, frequency, and mortality. A full abstract of this work will be found in a previous Number of this Journal.

*Acute rheumatism.* A somewhat dubious case of the transmission of acute rheumatism by a mother to her child, by suckling, is recorded by Dr. Gottfried Crusiz.§

*Fear, its influence on public health.* Dr. Zimmerman‖ has given a very interesting account of the influence exerted on the public health by the great fire at Hamburgh in 1842. He notices particularly the fact that many bedridden invalids rose and displayed supernatural force and energy, some of whom remained permanently cured. Diarrhœa, mania, and apoplexy were the principal diseases observed. There were 43 deaths, and 120 wounded. The monthly mortality was, however, below the average.

*Climate, meteorology, &c.* Numerous treatises have appeared on the influence of climate, &c. on the production and spread of disease, and the mortality attending it. Among others the following may be mentioned as containing much valuable information. Mr. Noble's** essay on the Influence of Manufactures upon Public Health; the Reports of Mr. Chadwick†† and the Registrar-General,‡‡ and of the Commissioners appointed to take the census of Ireland in 1841;§§ the treatise of M. Melier on the Influence of the Price of Provisions on general Disease and Mortality;‖‖ that of Dr. Guy, on the Influence of the Seasons and weather on Sickness and Mortality;*** Dr. Forry's treatise on Meteorology, &c.,††† and his analysis of the American army reports;‡‡‡ Dr. Foltz's essay on the Diseases

* New England Quarterly Journal of Medicine and Surgery, July, 1842.

† Provincial Medical and Surgical Journal, May 22, 1844.

‡ Recherches et Observations sur les Causes des Maladies Scrofuleuses, par J. G. A. Lugol; Paris, 1844.

§ Oesterreisch. Med. Wochens. 15 Oct. 1842.

‖ Oppenheim's Zeitschrift, Dec. 1843, p. 457.

** Facts and Observations, &c., by Daniel Noble; London, 8vo, pp. 88.

†† Report on the Sanitary Condition of the Labouring Population of Great Britain, &c., by Edwin Chadwick, Esq.; London, 1843, pp. 280.

‡‡ Fourth and Fifth Annual Reports of the Registrar-general.

§§ Report of the Commissioners appointed to take the census of Ireland for the year 1841; Dublin, 1843, folio: containing Mr. Wilde's 'Report on the Tables of Death,' founded on the mortality of Ireland for the ten years ending 6th June, 1841, amounting to about 1,187,374 persons.

‖‖ Etudes sur les Subsistances envisagées dans leurs rapports avec les Maladies et la Mortalité. Par M. Melier, in Mém. de l'Académie Roy. de Méd., t. x.

*** Quarterly Journal of the Statistical Society, on the influence of the seasons and weather on sickness and mortality, by Dr. Guy; and Medical Gazette, vol. xxxii.

††† Meteorology, &c. by Samuel Forry, M.D. 1843.

‡‡‡ The Climate of the United States, and its endemic influences; based chiefly on the records of the Medical Department and Adjutant-general's office. By Samuel Forry, M.D. New York. Langley, 1842. 8vo, pp. 380.

&c. of Minorca;* and that of Mr. Power, on the climate of Van Diemen's Land.

### 2. GENERAL DOCTRINES OF DISEASE.

*Systematic treatises on general pathology.* Dr. Schultz‡ has issued the first part of a systematic treatise on general pathology, founded chiefly on his own microscopic investigations in anatomy and physiology, and the peculiar views to which these have led him. An exposition of these views has already been given in this Journal,§ from which some notion may be gathered of the pathological doctrines propounded in the present treatise, which may be considered as a systematic attempt to apply to pathology the various important revelations of recent microscopic anatomico-physiological researches. The character of the work, however, precludes any abstract of it being given in a Report like the present. Dr. C. J. B. Williams's Principles of Medicine‖ will be found to contain an exceedingly valuable exposition of the general principles, not only of pathology, but also of general therapeutics. The work is alike conceived and executed in a far more philosophical spirit than anything which has issued from the British press for many years, with the exception, perhaps, of the invaluable Outlines of Professor Alison ; from the latter, however, the principles of Dr. Williams differ, in containing as an appendix to each section, the general principles of therapeutics, which are also discussed throughout the work in immediate connexion with the pathological doctrines whence they are deduced. Viewing disease as consisting in changes of function, or structure, generally of a more or less compound character, involving several elementary functions, or structures, he proceeds, as the anatomist or physiologist would do, to analyse and separate these derangements of structure and function, into their constituent parts, before contemplating them in combination. He treats, therefore, of general pathology by the synthetic method, commencing with the *primary* elements of function in its diseased state—viz. irritability, tonicity, sensibility, voluntary power, secretion, &c. Having considered the morbid changes in the vital properties of the elementary solids, he examines the morbid changes of the blood, and then passes to the consideration of the secondary or *proximate* elements of disease, consisting of two or more primary elements, commencing with those which relate to the circulation of the blood—anæmia, hyperæmia, inflammation, and its results. Structural diseases, or those of nutrition, are then discussed, and the chief forms of alteration in the nutritive processes traced, in which these diseases originate. The concluding chapters are devoted to nosology, semeiology, diagnosis, &c.

*Periodicity of disease.* Dr. Laycock adopts the term proleptics (from προλαμβανω, anticipo,) to designate the science of the laws of recurrence of phenomena, and has given his views of those laws in several "contributions to vital proleptics," in which he endeavours to exhibit the laws of vital periodicity, illustrating them by pathological phenomena, and showing how they may be applied to medicine. The causes of vital periodic movements he considers to be either esoteric, or exoteric. Schweig's†† researches on the same subject are intended to illustrate the periodic movements exhibited in the excretion of uric acid, in the uterine functions, in the number of deaths from various diseases, and in the recurrence of epileptic attacks. He terms the periods within which the changes which the body is continually undergoing in its composition take place, "tropical periods,"

* The Endemic Influence of Evil Government, illustrated in a view of the climate, topography, and diseases of the island of Minorca, &c. By J. M. Foltz, M.D. Surgeon in the United States Navy.

† Observations on the Climate of Van Diemen's Land, by W. J. Power.

‡ Lehrbuch des allgemein. Krankheitslehre, von Dr. C. H. Schultz, 1ter Thiel; Berlin, 1844.

§ July, 1843.

‖ Principles of Medicine, comprising General Pathology and Therapeutics, by C. J. B. Williams, M.D.; London, 1843. ** Lancet, 1842-3-4.

†† Untersuchungen über periodischen Vorgänge, &c. von G. Schweig; Carlsruhe, 1843.

(τρεφω, nutrio,) and adopts the quantity of uric acid excreted, as the exponent of the intensity of those changes. Instructions for the observation of periodic phenomena, and for obtaining uniformity of data have been published by Quetelet* and Schwann.† M. Melier‡ directs attention to the importance of recognising certain forms of disease in which he maintains the phenomena are intermittent; but with short intervals, and instances certain convulsive affections of infants, some cases of eclampsia, uterine pains, hemorrhages, and fluxes, in which the phenomena of intermittence may be observed, and which cede to the influence of bark. M. Duparcque§ also confirms the observations of Melier, and cites similar cases in support of these views. He relates among others a case in which paroxysms resembling those of ague occurred four times in the twenty-four hours, with almost perfect intermissions, and which were cured by sulphate of quinine—two cases of convulsions in infants—and hiccup in old people. He suggests that certain mental affections belong to this category, and gives a case of acute intermittent delirium, with short intervals, cured by quinine.

*Antagonism of disease,—phthisis and ague.* Numerous and warm discussions have taken place in the Royal Academy of Medicine, and other scientific societies of France, on a statement made with considerable confidence, by M. Boudin, in his 'Géographie Médicale,' to the effect that a real antagonism exists between phthisis and ague, so that in any district where the one is a frequent disease, the other is rare. To this opinion he had been led by his observations on the diseases and medical topography of Algiers, where, he says, the "rarity of phthisis is not to be considered as a general fact, but as true only with reference to the marshy part of the coast where intermittents and other diseases from malaria prevailed;" hence he infers that the phthisical and those disposed to phthisis should reside where the temperature is mild or warm, and the soil marshy, though not otherwise very unhealthy. In support of his opinion he cites Hyères, long known to be favorable to the phthisical, though liable to malarious diseases,—Pisa, Plaisance, Parma, and Rome. He refers also to the statement of Hennen, with reference to the rarity of phthisis and frequency of intermittent diseases in the British isles of the Mediterranean, and to numerous other writers whose evidence is to the same effect. M. Boudet, when on the point of proceeding to Algeria, was requested by the French Academy to endeavour "To determine whether phthisis is a rare disease in Algeria, and whether it is true that it is much more rare in the marshy districts than in other localities."‖ This subject of inquiry the academy recommended in consequence of the above statements of M. Boudin and those of M. Casimir Broussais, who, in a previous memoir, read to the Academy, asserted that he had ascertained from the official army reports, that the proportion of deaths from phthisis in Algeria, to those from other diseases, was 1 : 102, while in the army in France the proportion is 1 : 5. The reporters on this memoir, however, with much reason, doubt whether this rarity of phthisis, as stated by Broussais, is not more apparent than real, and may not be explained by the great mortality of the army from other causes. From various sources, a mass of evidence has been collected, with a view to determine the truth of Boudin's theory, in favour of which may be cited the evidence of M. Nepple of Lyons,** who states that the rarity of phthisis in marshy districts, "has always appeared to him in direct relation to the elements of 'impaludation,'" and to diminish with them. "So that if in the districts situated in the centre of a swampy country we do not meet with a single case of indigenous phthisis, we find that the number of cases increases in proportion as we recede from that district. Hence, at a certain point we find tubercles and ague associated; but, then the febrile endemic is slight." The evidence of Pacord, of Bourg (en Bresse) and of others is to the same effect.††

* Bulletin de l'Acad. Roy. de Bruxelles, No. i, t. ix. † Idem, Nos. i and vii, t. ix.
‡ Bulletin de l'Acad. Roy. de Méd., 15 Mai, 1843. Rapport by F. Dubois, &c.
§ Gaz. Méd. de Paris, 24 Dec. 1842. ‖ Bullet. de l'Acad. Roy. de Méd. No. xvii, 15 Mai, 1843.
** Gazette des Hôpitaux, Sept. 2, 1843. †† Idem, loc. cit.

But the evidence adduced by other observers is directly opposed to the theory of Boudin; and the results of Dr. Genest's examination of the reports of Major Tulloch and Mr. Wilson on the sickness and mortality of the British army at home and abroad seem decisive against the theory, as will appear from the following table, showing the relative number of cases of phthisis and intermittent fevers admitted into hospital.*

| | Intermittent Fever. | Phthisis. |
|---|---|---|
| United Kingdom | 2 per 1000 | 6·5 per 1000 |
| Gibraltar | 5 ,, | 5·6 ,, |
| Malta | 7·5 ,, | 6· ,, |
| Ionian Isles | 132 ,, | 5· ,, |
| Canada | 78 ,, | 5·6 ,, |
| Nova Scotia | 0·8 ,, | 7· ,, |
| Bermudas | 2·5 ,, | 8·8 ,, |
| Western America | 250 ,, | 9·5 ,, |
| Jamaica | 85 ,, | 13· ,,† |

*Purulent infection, Theory of.* M. Sedillot,‡ after pointing out the insufficiency of the various theories advanced to explain the phenomena of purulent infection or metastatic abscesses, endeavours to show that as long as pus is of the healthy kind, it exerts no toxical influence on the economy, and proceeds to inquire what are the alterations of quality necessary, to give rise to visceral abscesses and adynamic fever. Changes from simple exposure to the air are not sufficient, but it is not necessary that the pus should be sanious or fetid. The principal causes of the phenomena of purulent infection he believes to be the gangrenous or ulcerative liquefaction (*fonte ulcereuse*) of parts in a state of ulceration, a condition which he has always found to exist, and which is occasioned by the pressure of pus on the surrounding tissues and their consequent death from strangulation, which precedes the absorption of their detritus. Dr. Budd's valuable lectures on suppuration of the liver contain many important facts bearing on the same subject.§ He arranges the causes of suppurative inflammation of the liver under three heads: 1st, mechanical violence; 2d, suppurative inflammation of veins—the common cause of disseminated abscess after operation; 3d, ulceration of the intestines, stomach, and gall-bladder, but especially of the large intestine. This he considers the most frequent cause, and adverts to the connexion between dysentery and abscess of the liver as having been long known, but which he thinks more frequent than has been supposed. In thirty-four of fifty-nine cases which he cites, this connexion existed; the same proportion existed in twenty-nine cases given by Annesley; and in fifteen from Louis and Andral. It is chiefly in connexion with the sloughing ulceration of acute dysentery and chronic ulcers that hepatic abscess is seen. A close analogy, Dr. Budd thinks, exists between disseminated abscesses and disseminated masses of cancer, from the absorption of cancer-cells. The experiments of Mr. Betts|| [analogous to those of Dance and Cruveilhier,] are confirmatory of Dr. Budd's views, showing that the injection of mercury and pus by the blood-vessels will produce inflammation and abscess of the tissues and organs, supplied by the capillaries in which these vessels terminate. Pus injected into the mesenteric vein induced abscess of the liver, and when injected into the crural or other systemic veins, abscess of the lungs. Pus-globules and cancer-cells, Mr. Betts states, he has ascertained by admeasurement to be too large to pass the capillaries, where consequently they are arrested and give rise to irritation.

*Tubercle.* The conclusion to which Mr. Addison's researches on tubercle** have led him is "that all secretions take place in the interior of granulated vesicles

* Gazette Médicale, Sept. 9, 1843.

† Vide also Gazette Méd. de Paris, July 1, 1843. Idem, Aug. 5. Gaz. des Hôpitaux, Nov. 9, 1843. Idem, Aug. 31; and Bulletin de l'Acad. Roy. de Méd. Scèance, Aug. 29, 1843.

‡ Annales de la Chirurgie, Franc. et Etrangère, t. vii, p. 129.

§ Gulstonian Lectures, by Geo. Budd, M.D.; Med. Gazette, vol. i, 1843, pp. 1, 33, and 65.

|| Medical Gazette, vol. i, 1843-4, p. 312.

** Researches on the Nature of Tubercle, Med. Gazette, Nov. 11, 1842.

or cells, not by transudation from one tube (a blood-vessel,) into another, (a duct,) and consequently that "tubercles' in the lungs, "tubercular infiltrations," "hepatization," and "pus" are not secreted products, but simply the elements of the blood effused by an excessive "vital turgescence," (or inflammatory action,) having their peculiar characters determined by the texture and function of the structures, and the amount of activity of the turgescence. As far as can be determined by the microscope, the substance effused in pneumonia differs not from tubercular matter. Dr. Lebert, however, maintains* that gray granulations (which he says are tubercular) and tubercles, are proved by the microscope to be no product of inflammation. The constant microscopical elements of tubercle are molecular granules, an inter-globular hyaline substance, and certain corpuscles or globules peculiar to this abnormal product. Neither is tubercle merely a modification of pus. Tubercles of the lung are usually seated in the intervascular cellular tissue, but occasionally in the cells and the minute bronchi. The views of Dr. Schultz† appear in the main to coincide with those of Lebert. He contends against the views of Gluge, Vogel, and others, and says that cells are not essential to tubercle, but occur only in its latter stages, and indicate in fact the formation of pus-globules. M. Briquet‡ relates three cases of tubercular disease, in support of the following propositions. 1. That there are cases in which the tubercular or cancerous diathesis is primary, and in which these heterologous productions are developed on the surface of serous membranes, without having previously existed in the principal viscera. [The two cases which he adduces in support of this proposition which is opposed to the law laid down by Louis, would scarcely be considered satisfactory by that distinguished pathologist, inasmuch as some tubercles in both cases existed in the lungs. But more decisive cases might easily be adduced, for the exceptions to the law of Louis are certainly numerous. Some will be alluded to in the second division of this Report under the head of strumous peritonitis.] M. Requin§ communicated to the Société Méd. de Paris, the case of a young man æt. 17, who died from extensive tubercular disease of the mesenteric glands, the lungs presenting no trace of tubercle. Briquet's third case is an example of extensive encephaloid disease of the peritoneum attended by inflammation of that membrane, and of the pleura, without any disease of the viscera. His 2d proposition is, that inflammation of the serous membranes, gives rise to the exudation of a matter which passes immediately and without transformation, to the state of tubercle or encephaloid granulations. The situation of the deposits, and their connexion with other indications of inflammation, are the chief arguments he adduces in support of this proposition. 3d. The dropsy which ordinarily accompanies inflammation of serous membranes, that is productive of heterologous matters, may be diagnosticated by the peculiarity of its symptoms and progress. [The evidence advanced in support of this proposition, is only sufficient to establish the distinction between general dropsy from visceral disease or venous obstruction, and that which depends on the accumulation of fluid from chronic inflammation.]

M. Rayer‖ who has, for many years, been engaged in the study of comparative pathology, has given, in the following among other propositions, the results of his inquiries on tubercular phthisis. 1. In man and other mammifera, the matter of tubercle may be readily distinguished from recent pus by the absence of granular globules. In birds, the characters of tubercular matter are less marked; foreign bodies introduced into the lungs or flesh, do not give rise to the exudation of a white opaque fluid containing granular globules, but to a dry yellowish matter destitute of globules, the physical characters of which, approach to those of tubercles in the mammalia. In reptiles, fishes, and insects, the characters of tubercle are still less distinct. 2. Pus in the mammifera, es-

* Comptes Rendus, No. x, 1844.
† Lehrbuch, &c.
‡ Archives Gén. de Méd. Oct. 1842.
§ Revue Méd. Sept. 1842.
‖ Journal des Connaissances, Medico-Chirurg. Aug. 1, 1842.

pecially in the horse, after long residence in the organs of the body, undergoes a series of transformations, in consequence of which, it sometimes acquires the appearance of tubercular matter. 3. In man and animals the central softening of tubercles never presents pus-globules, and cannot be ascribed to inflammation; the peripheral softening on the contrary, is generally produced by inflammation of the contiguous textures, and is almost always mixed with pus-globules. 4. The cretaceous or calcareous concretions, (principally composed of carbonate and phosphate of lime with animal matter,) observed in the lungs of both man and animals, must not be considered, as they generally have been, as the last modifications of tubercle, for they are often in man, and very often in the horse, the residue of a small deposit of pus. 5. In many animals granulations are developed in the lungs, which are due to worms or glanders, and which may be mistaken for tubercles. 6. Phthisis attains its maximum frequency, in the quadrumana and birds brought from warm climates, and is likewise favoured by the change in climate and food of animals coming from cold climes, as in the rein-deer. 7. Phthisis is rare in the domesticated solipeda, and still more so in the carnivora, but many of the latter are attacked. 8. The domestic dog among the carnivora, and the horse among solipeda, are much less liable to tubercle than to cancer, a disease that had been thought by Camper to be alien to these animals. 9. Among the ruminantia, especially in the genus ox, phthisis is often associated with vesicular entozoa, (especially the ecchinococcus,) but there is no relation of transformation or succession between the hydatids and tubercles. 10. Fatty degeneration of the liver is ordinarily an indication of phthisis in man, and of obesity in birds. 11. Although the frequency of pneumonia and rarity of phthisis in the domestic dog seems to show that these diseases are independent of each other in their occurrence, nevertheless in the calf, milch-cow, and ass, the deposits of tubercular matter almost always coincide with a chronic progressive pneumonia. 12. Ulcers of the larynx, trachea, and bronchi have not the same import in all animals as in man; in the quadrumana they almost always indicate a general tubercular affection; in the solipeda almost always glanders. 13. In pneumo-thorax, growths of vegetable fungi may be found on the diseased pleura of phthisical persons, in the same way as they are sometimes found in the air-sacs of birds, but in all cases the development of these fungi is a secondary phenomenon.

*Tubercles in the bones.* M. Parise* describes the appearances presented in a patient in whom tubercles were found in the lungs, spleen, cellular tissue, and the spinal canal, and in the substance of the vertebræ and sacrum. In the latter situations were seen, 1st, an isolated tubercle surrounded by a vascular membrane, and inclosed in an osseous cell, the surrounding cells being healthy: 2, gray semitransparent matter infiltrated in the bony tissue: 3d, purulent infiltration and necrosis. The author concludes that necrosis is the result of distension of the osseous cells from the infiltration of the yellow tubercular matter.

*Carcinoma.* Dr. Hodgkin,† in pursuance of his former investigations, has endeavoured to connect the nucleated carcinomatous cells of Müller, with the production of those compound cysts, which Dr. Hodgkin had formerly described and pointed out, as the type of the carcinomatous group of adventitious structures. Microscopic investigations have confirmed his views, and demonstrated the application to them of the nucleated cell-theory; whilst these investigations are fatal to the theory of cancerous matter being formed in the blood, and eliminated at the spot where the tumours appear. An additional argument is thus afforded in favour of operations, and the importance of removing every cyst rendered more manifest.

*Nervous diseases, Theory of.* Dr. Wilson has advanced an hypothesis‡ with reference to various diseases affecting the muscular system, and usually termed

* Archives Gén. de Médecine, 1843, p. 208. † Medical Gazette, June 24, 1843.
‡ On Spasm, Languor, Palsy, &c. &c. by James A. Wilson, M.D.; London, 1843.

nervous, which induces him to consider general spasmodic as well as paralytic disorders, in the greater number of cases, to be induced by the direct morbid influence of certain agents circulating in the blood, upon the muscular fibre, rather than upon the nervous system.

### II. Special Pathology.

1. Diseases of the Digestive System.—*Œsophagus.* An example of greatly dilated œsophagus is recorded by Professor Huss, of Stockholm.* A lady, æt. 43, had for many years regurgitated her food (often six or twelve hours after taking it) unaltered, and been the subject of hysteric spasms. After death, dilatation of the œsophagus was observed, commencing one inch below the pharynx, and gradually increasing till, at the cardia, it formed a large sac lying to the left of the stomach, which was small, but there was no obstruction at the cardia, nor any structural alteration of the œsophagus, except considerable thinning. Dr. Huss attributes the dilatation to the hysterical spasms. —A case of cancer of the œsophagus opening into the right lung, is recorded by Dr. Jackson.†

*Stomach, perforating ulcer of.* Mr. Crisp‡ has related 5 cases of perforation of the stomach, and collected 46 others from different sources; and from a survey of the whole draws the following conclusions. Of 51 cases, 12 were males and 39 females, and the ages of the latter as follows.

| | |
|---|---|
| Between 15 and 20 | 21 |
| ,, 20 and 25 | 10 |
| ,, 25 and 30 | 5 |
| ,, 40 | 1 |
| ,, 50 | 1 |
| ,, 60 | 1 |

Most, if not all, of the females were unmarried, and in 13 the menses were irregular. Perforation from simple ulcer, he states, rarely, if ever, occurs before puberty in females, seldom after the cessation of the menses, and almost invariably in the unmarried. A chlorotic condition of system, he thinks, is the predisposing cause. In the majority of cases the opening was situated in the smaller curvature, and generally midway between the pyloric and cardiac orifices; in one instance only, it was close to the pylorus; in nine, two ulcers were present on opposite surfaces of the stomach, so that when collapsed the diseased parts were in contact. Seven cases are also detailed by Dr. A. Lefèvre,§ who has succeeded, on the dead body, in rupturing the stomach by excessive inflation, and states that the openings thus made are of various forms, some *perfectly round.* He believes many of the instances of sudden perforation are caused by the ingestion of indigestible food, and the generation of gas; that the stomach is thus paralysed, and unable either to pass its contents, or expel them by vomiting; that the acrid mass being retained softens the stomach, and thus further disposes to perforation. In a case detailed by Dr. Morici,|| the patient, a male, who was recovering from intermittent fever, and had had no previous gastric symptoms, died suddenly on leaving the water-closet. The stomach was found torn along the middle of its anterior surface to the extent of three fingers' breadth. The only other morbid appearance being a little thickening of the mucous membrane along the edges of the aperture.

*Ileum, perforation of, from without.* A rare case of perforation of the ileum from ulceration of the peritoneum, consequent on chronic peritonitis, is recorded by Dr. A. M. Adams.** The peritoneum was ulcerated in two places over the commencement of the ileum, and one of these ulcers had perforated

* Gazette Méd. de Paris, Feb. 11, 1843.
† New England Journal of Med. and Surg. Oct. 1842.
‡ Lancet, Aug. 5, 1843.
§ Archives Générale de Méd. Sept.
|| Annali Universali di Medicina, April, 1844.
** London and Edinb. Monthly Journal, Jan. 1844.

the bowel. The corresponding portion of mucous membrane was perfectly healthy.

*Cæcum. Perforation of the appendix vermiformis.* Numerous cases of this kind have been recorded; six by M. Valtz, of Carlsruhe,* who, comparing these with fourteen from other sources, attempts to lay down rules for the diagnosis of peritonitis from this cause. He finds the accident to be much more common in males than in females; of 20 cases 17 being males. Two cases are given by Dr. Ernst, of Bonn,† in which hard fecal masses were found in the appendix. In a case recorded by Dr. Peebles,‡ a mulberry calculus of very irregular surface, and nearly an inch in diameter, was found impacted in the appendix. The nucleus consisted of organic matter, and to the surface were attached a number of tomato seeds. The composition of the calculus was not ascertained, but from the history it appears to have been lodged in the appendix for twelve years. Mr. Worthington, of Lowestoff,§ relates a similar case, occurring to a boy, æt. 11, who died after three days' illness with symptoms of acute enteritis. The appendix was perforated near the extremity, and further towards the cæcum an oblong calculus, of a gray colour, and three-quarters of an inch long, was found "so beautifully impacted" that no analysis was made. In Mr. Butler's case (of Winchester),‖ the patient, a boy, had suffered from symptoms of gastric and biliary derangement, with fever and pain of the right thigh and groin, increased on pressure, with tenderness of abdomen and constipated bowels, and died from peritoneal inflammation. The appendix cæci was enlarged, thickened, and of a dark colour. In its upper half were two circular perforating ulcers, through which had escapedtwo small bodies of the size of large peas, of a light brown colour, and nodulated surface, and which corresponded with a third body found in the appendix. The intestinal canal and gall-bladder were free from calculi. On analysis, the calculi were found to consist of inspissated mucus, fatty matter, and a small proportion of oxalate of lime. This analysis corresponds to that in Mr. Wickham's case (Lond. Med. Journal, vol. 3,) with the exception that in the latter there was a portion of subphosphate of lime. From this it would appear that the calculi are not of biliary origin, but formed within the appendix, the natural secretion of which is acid.**

*Gall-bladder enormously distended.* Dr. Babington†† has recorded an example of enormously distended gall-bladder, the cyst containing at least three wash-hand basins of fluid.

*Colica pictonum.* A fatal case of this disease is detailed by Mr. Wreford, of Ottery St. Mary,‡‡ The patient died on the sixth day. The colon was found irregularly contracted, and presented several ecchymosed spots. The appendix cæci was in a state of gangrene.

*Spleen.* Signor Verga§§ details the case of a woman, æt. 50, who, having three years previously received a blow on the abdomen from the pole of a carriage, became subject to attacks of abdominal pain, with obstruction of the bowels, from which she untimately died. In the left iliac fossa was found a tumour, presenting the characteristics of the spleen, surrounded and hidden by numerous adhesions. The spleen was wanting in its natural situation, and the splenic artery obliterated towards the middle of its course, where it terminated in a point. Cases of spontaneous bursting of the spleen are recorded by Dr. Alle‖‖ and Dr. Neill,*** and an instructive case of chronic enlargement by Dr. Davis.†††

* Archiv. für die gesam. Med. No. iii, t. iv, 1843.

† Schmidt's Jahrbücher, No. iii, 1844, p. 285.

‡ American Journal of Med. Sciences, Jan. 1843.

§ Prov. Med. and Surg. Journ., April 8, 1843.

‖ Dublin Medical Press, April 5th, 1843, from Provincial Medical and Surgical Journal.

** Observations on this subject will also be found in the following papers: Durchbohrung des Wurmfortsatzes, von Dr. Mohr, in Casper's Wochensch. No. xlii, 1842, and Mai 20, 1843, and in Hufeland's Journal for March 1843, by Dr. Binger.

†† Guy's Hospital Reports, vol. vii.

‡‡ Prov. Med. and Surg. Journal, July 15, 1843.

§§ Gazetta Medica di Milano.

‖‖ Oesterreisch. Med. Wochensch. Dec. 17, 1842.

*** American Journal of Medical Sciences, Oct. 1842.

††† Ibid.

2. Diseases of the Circulatory System.—*Heart. Carditis.* A remarkable case of partial carditis, terminating in abscess and fistulous communication with the pericardium, is detailed by M. Gintrac, of Bordeaux.* A man, 68 years of age, had for one year been subject to palpitations, which, after a violent fit of passion, became much more severe, and were attended by orthopnœa and anarsarca of the lower extremities. When he entered the hospital the heart's action was violent and irregular, and heard over a large extent of surface, but no preternatural sound could be detected, perhaps owing to the loud sonorous ronchi which accompanied the respiration. The pulse was small and frequent, the skin cold, and the countenance expressive of great anxiety. The pericardium was found surrounded by a thick layer of fat, and contained a turbid reddish fluid, resembling a mixture of pus and bloody serum; the serous membrane was slightly reddened. The heart was large, and its anterior surface covered by a layer of concrete pus; over the left ventricle the serous membrane was opaque and easily detached, and at this spot an oval aperture was seen passing from above downwards. The inferior portion of the cavity of the left ventricle was separated from the upper by a thick unorganized layer passing from one side to the other, and forming a sort of septum, beneath which was a collection of thick purulent fluid of the colour of wine lees, in which were masses of a thick solid substance resembling altered clots of blood. The muscular substance corresponding to this abscess, was soft and of a grayish colour; anteriorly the muscular fibres were infiltrated with pus, and from this point a fistulous canal was traced upwards, and found to communicate with the external aperture already described. The membranous septum separating the inferior from the superior portion of the ventricle, appeared to have completely prevented the blood from mixing with the contents of the abscess, and from escaping by the fistulous opening into the pericardium, notwithstanding the forcible action of the heart. —Dr. Dubini also gives the following case.† A woman was admitted into hospital after four days' illness, with pyrexia, dyspnœa, weight in the precordial region, double bruit, rough and superficial, extending along the course of the aorta, and the beat of the heart not free. Subsequently, there was feebleness of the heart's sound and absence of pulse. The muscular substance of the heart was of a yellow colour, and infiltrated with pus; the aortic valves insufficient, and the apex of the heart bound down by adhesions to the pericardium. A similar case is referred to, as occurring four days later in the same hospital, with more advanced purulent infiltration.

*Endocarditis confined to the right side.* Dr. Burci, of Florence,‡ details a case of extensive bronchitis, accompanied with rheumatic pains, and presenting after death evidence of endocarditis, confined to the right auricle, which was filled with liquid blood, mixed with abundant albuminous flocculi. The endocardium was of a florid vermilion colour, tumid and undulated. Layers of dense false membrane covered those parts of the endocardium which were reddest, and beneath which it was thickened, opaque, and readily torn. No other morbid appearance existed, except that the heart was large, flaccid, and soft. Dr. Graves§ found the valves of the pulmonary artery (of which there were but two) studded with recent lymph, in a man who died in 26 hours after a sudden aggravation of symptoms occurring in the course of pneumonia of the right lung.

*True organised polypus of the left auricle.* The following almost unique case is described by M. Puisaye.‖ A young man, æt. 19, who from eight years of age, had been the subject of cardiac symptoms, but had never suffered from rheumatism or any acute affection, complained of an almost continued sense of suffocation. There was violent impulse of the heart felt over a great extent of surface, with precordial dulness. The first sound was accompanied by a loud harsh blowing, having its maximum intensity at the apex of the heart,

* Bulletin de l'Acad. Roy. de Méd. April 11, 1843.
† Gazetta Medica di Milano, Jan. 1844.
‡ Ib. Oct. 14, 1843.
§ Dublin Journal of Medical Science, Jan. 1843, p. 398.
‖ Gazette Médicale de Paris, April 29, 1843.

and which was lost on approaching the aorta; pulse 92, small, irregular. Diarrhœa and gastric disturbance, succeeded by hemoptysis, weret he precursors of death. General hypertrophy and dilatation of the heart were discovered, and the left auricle was distended by a red tumour, having, at first sight, the appearance of a clot of blood, but which on examination proved to be a lobulated fungoid tumour of the consistence of jelly. It distended the auricle and passed through the mitral valve into the ventricle. Its attachment was to the auricle near the foramen botale, around which the lining membrane was puckered up, in folds, which passed into the tumour, and formed its pedicle, the attachments of which were so firm as to admit of the whole organ being suspended by the tumour. The base of the tumour was of cartilaginous hardness, but the body consisted of a number of soft lobules, attached like grapes to the central stalk.

*Needles in the parietes of the heart.* Dr. Sklarsky,* a Russian physician, relates a case of aneurism of the aorta occurring in the person of a woman, æt. 50, and proving fatal by rupture into the pericardium. On examination, a sewing-needle one inch long, was found so firmly imbedded in the substance of the right auricle, and so corroded that it broke into several pieces on attempting to extract it. Dr. Sklarsky supposes that the needle having been swallowed, stuck in the œsophagus, then passed into the aorta, and gave rise to the aneurism, whence by the movements of the heart, it was thrust into the auricle. In the following case recorded by Dr. Leaming,† the progress of the needle appears to have been traceable by the symptoms. A young woman, when stooping over a table, ran a needle into the right breast; a month subsequently she was suddenly seized with pleuritis, after stooping to pick something from the floor. Five months after this, she had pneumonia, with bronchitis of the right lung, and within another month spasms of the diaphragm, which were succeeded by obstinate vomiting and subsequently by pain about the heart and pericarditis. The needle was found after death in the heart, passing from the back, through the right ventricle into the left.

*Heart, aneurisms of.* In a lengthy paper on this subject,‡ Dr. Craigie has collected from various sources, twenty cases of aneurism of the heart. He calls attention to the dilated recesses or pouches between the columnæ carneæ, near the apex of the heart, which are not unfrequently seen in dilatation of the left ventricle, and in some cases of hypertrophy, and which are usually filled with adherent coagula, as though the blood had been, for some time, out of the current of the circulation. These dilated recesses, he thinks, must be regarded as incipient aneurismal dilatations. They are frequently associated with attenuation of the apex of the heart. He details a well-marked case of partial aneurism of the heart, that fell under his own observation, remarkable from the situation of the dilatation and destruction of parts, being at the base of the septum cordis. In the septum cordis in the space between the right lacinia of the mitral valve, and two of the semilunar valves, was a large oval aperture, leading into a cavity of a spherical form, sufficiently large to admit and contain a good sized walnut. The walls of this cavity formed a projecting tumour into the right ventricle, the dimensions of which, at its base were thus considerably diminished. The margin of the tumour or cavity was distinct and sharp, forming a sort of collar to the tumour, as well as the aperture of communication between the cavity of the ventricle, and that of the sac, which was filled, not with solid lamellar adherent coagula, but with semifluid, grumous blood. The walls, more particularly of the convexity protruding into the right ventricle, were composed chiefly of *fibrous* bands.

Mr. Hodgson§ of Birmingham, has also related a case in which there was great distension of the apex of the left ventricle, so as to form a pouch nearly as large

* Oesterreisch. Med. Wochen. 1843, p. 464. † Philadelphia Medical Examiner.

‡ Observations and cases illustrating the nature of false consecutive aneurisms of the heart, by D. Craigie, M.D. Edinburgh Medical and Surgical Journal, April, 1843, p. 357.

§ Provincial Medical and Surgical Journal, Dec. 9, 1843.

as the natural cavity, the sac was filled with coagula and layers of fibrin, as in ordinary aneurism. The parietes of the heart at the diseased part, were so attenuated, that little or no muscular structure could be seen. Mr. Hodgson thinks this is the most frequent seat of the disease, and that, imperfect nourishment—conversion into tendinous substance—and the deposition of tubercular matter, are the chief causes.

*Heart, tubercles of.* Dr. Kerst* records the case of a young man, æt. 21, who died much emaciated, but without any symptoms exciting suspicion of cardiac disease. Tubercles were found in the upper part of the left lung and mediastina, the heart of usual size, was throughout adherent to the pericardium, between the two surfaces of which were large masses of tubercle; two large tubercles existed in the muscular tissue, all in a state of softening, and yellow softening of the outer wall of the left ventricle.

*Communication between the right and left side of the heart without cyanosis.* In one case of this kind, given by Professor Huss of Stockholm,† a communication existed between the pulmonary artery and the aorta, large enough to admit the ring finger. Palpitation had been the only cardiac symptom. In another instance recorded by Dr. Mayo,‡ the patient, a woman æt. 57, had been the subject of chronic bronchitis, accompanied by sudden accesses of dyspnea, small quick pulse, increased cardiac impulse, and dullness with a loud systolic bruit at the apex. The foramen ovale was open to the extent of one inch and a quarter.

*Heart, varix of.* Dr. Albers§ gives two cases of sudden death from the rupture of greatly distended coronary veins, the patients having been the subjects of asthmatic symptoms. Such cases he has found associated not with true hypertrophy of the heart, but with either a normal or thinned condition of its walls and the deposition of much fatty matter.

*Diseases of the orifices and valves.* By the careful examination of a large number of cases, Dr. N. Chevers‖ has endeavoured to ascertain what are the abnormal conditions of the orifices and valves which are either unimportant in the production of morbid symptoms, or are the results of the means adopted by nature to limit the extension of disease, and adapt the diseasedheart to its altered circumstances.

*Obliterated and contracted aorta.* Dr. J. Hamernjk¶ has detailed at great length a case in which the aorta was completely obliterated immediately after giving off the left subclavian. The patient had, some years before his death, been squeezed by a waggon, and presented during life, a number of pulsating tumours along the back, consisting of a varicose and greatly enlarged condition of the branches of the arteriæ transversalis colli, scapulæ and subscapularis. The internal mammary arteries were also found, after death, greatly dilated, and the left subclavian at its origin six and a half lines in diameter. A similar case is given by Mr. Muriel,** in which the aorta near its junction with the ductus arteriosus was constricted almost to obliteration, and the superior intercostals much dilated.

*Vena Azygos, rupture of. Sudden death.* Dr. J. Flögel†† relates the case of a soldier who on parade fell suddenly from his horse, and died instantly. With the exception of the liver, all the organs were sound. But in the posterior mediastinum four pounds of black loosely-coagulated blood were found, which had escaped from a large rent in the vena azygos, which was enlarged throughout.

3. Diseases of the Respiratory Organs. *Larynx, Trachea, &c.* Dr. Stokes‡‡ has related a case of fatal chronic disease of the larynx, without pulmonary disease, or the usual symptoms of mechanical obstruction to the respira-

* Brit. and For. Med. Rev. Oct. 1843, p. 385. Waarnemingen in het Gebied der Path. in Utred, 1840.
† Gazette Méd. de Paris, Feb. 11, 1843.
‡ Lon. Med. Gazette, 1843-4, vol. i. p. 613.
§ Schmidt's Jahrbücher, No. 4, 1844, p. 37.
‖ Guy's Hospital Reports, vol. vii.
¶ Oesterreich Med. Wochen. March 4, 1843.
** Guy's Hospital Reports, vol. vii.
†† Oesterreich Wochensch. No. xi, 1844.
‡‡ Dub. Journ. of Med. Science, March, 1844.

tion. The disease was singularly localized, occupying the glottis alone, the orifice of which was completely closed by warty excrescences growing from the edge. Two examples of polypoid growths in the trachea have been recorded. In one, detailed by Mr. Stallard,* death occurred after a severe paroxysm of coughing, lasting an hour. A loose polypus of the size of an almond was found in the trachea, and the root of the pedicle was attached to the mucous membrane, just below the cricoid cartilage. The woman had, for some time, been subject to cough and asthmatical symptoms. In the second case, M. Ehrmann,† having ascertained the existence of a fibro-cellular tumour engaged in the rima glottidis, had recourse to tracheotomy to save the woman from impending suffocation, and two days subsequently cleft the thyroid cartilage, when the polypous excrescence was exposed, and removed from the inferior ligaments of the larynx, to the whole length of which it was attached.

*Pneumonia.* Dr. Addison‡ thinks that what is called simple pneumonia is not really so uncomplicated an affection as is supposed, but should rather be called a broncho-pneumonia. A truly simple pneumonia does, however, occur, and may be unattended by either cough, expectoration, or pain. He considers the air-cells to be the original seat of pneumonia, and not the interstitial tissue, of which he denies the existence. [In what way, then, does the author suppose the vessels which ramify on the exterior of the air-cells, are connected with the parietes of opposite cells; do they lie in naked apposition with the cells? There is surely, as all analogy would lead us to believe, some connecting tissue, and this, to other observers, at least, the microscope appears to reveal.] The first effects of inflammation are the arrest of the natural secretions of the cells, the effusion of serum into them, (not into the interstitial tissue,) subsequently the swellings of their walls, and thus encroachment on their cavities, and absorption of the serum previously effused. In this state the tissues are brittle and consolidated; in the state of red hepatization. As the inflammation proceeds, the parietes of the cells become more opaque and thickened, the minute blood-vessels are no longer visible, the tissues become soft, and with this loss of cohesion and diminished vascular turgescence, the cells admit of albuminous matter being poured into their cavities. This constitutes the gray hepatization. In cachectic subjects this change is commonly limited to separate lobules. What is called carnification of the lung, Dr. Addison considers to be merely the effect of pressure, sufficient to force out all the contained air. The above may be considered the immediate effects of inflammation. The permanent effects present three forms: 1. The uniform albuminous induration, which is the least frequent, but occasionally occurs as the result of acute pneumonia in healthy constitutions. The effect of this is to transform the parts implicated into a uniform, homogeneous, opaque, or semitransparent mass, in which no trace of the ordinary structure of the lung can be detected. 2. The granular induration, which is caused by the effusion of a less organizable albumen. In this form the lobules, with their still distinct cells, filled with the effused matter, may be distinguished, and present something of the character of a raspberry. This is sometimes called inflammatory tubercle. 3. The gray induration, consisting of a mixture of yellowish, white, and black matter in varying proportions, the density increasing with the darkness of the colour. The albuminous effusion, in this condition of lung, has partially undergone organization and contraction and thus glued together, and hardened the aërial cellular tissue. In proof of these changes being the result of inflammation, and not of tubercular infiltration, Dr. Addison adduces various [and satisfactory] reasons,

*Gangrene of the lung.* [This is by no means of so unfrequent occurrence as some have supposed, and the great interest and importance of the subject, as well as the complete obscurity in which the genesis of some forms of pulmonary

* London Medical Gazette, 19 May, 1843.

† Comptes Rendus, April 1, 1844.

‡ Guy's Hospital Reports, 2d series, No. ii.

gangrene is involved, will justify a brief notice of those cases which have been recorded.] Dr. Scharlau* has given a well-marked instance of idiopathic gangrene of the right lung. The patient, a robust man æt. 45, in the course of a slight attack of gastric fever, had cough and fetid breath, which led to a careful examination of the chest. Nothing abnormal could be detected by physical examination, though the character of the expectoration, as well as the insupportable stench of the breath, subsequently rendered the nature of the case clear. Shortly before death there was evidence of pleuritis of the right side. The post-mortem examination revealed a gangrenous cavity in the middle of the right lung, capable of containing two fists, but without any trace of pneumonia or tubercle. The pulmonary tissue, in the immediate vicinity of the gangrenous cavity, was permeable to air. The opposite lung was quite healthy.

In the case related by MM. Richard and Deschordes† the only indication of any pulmonary disease was the fetor of the breath, notwithstanding repeated careful examinations. The case, however, subsequently became complicated in a remarkable manner, from the sudden occurrence of peritonitis, having the characters of that arising from perforation of the intestinal canal, and attended by great tympanitis. After death it was discovered that a communication had taken place between a gangrenous cavity in the lung and the peritoneum. The details of the appearance presented by the lung are not given, the reporters dwelling only on the obscurity of the case during life, and its occurrence in a strong healthy young man, aged 21. In one of Mr. Wells's cases,‡ (occurring in a healthy man, æt. 28,) there appeared reason to believe that the gangrene was consequent on the pressure exercised by enlarged bronchial glands on the root of the lung. A great portion of the upper part of the right lung was hepatized and reddish black, and the centre of the upper lobe converted into a semi-pulpy mass, in which shreds of pulmonary tissue and obliterated vessels were floating. The opposite lung was œdematous and emphysematous, and the bronchial and mediastinal glands much enlarged. In his second case, (a marine of robust habit, æt. 26,) a portion, the size of a walnut, of the lateral surface of the lower lobe of the right lung was in a state of gangrene, the rest of the lobe being hepatized. In this and the former case, communication had taken place with the pleural cavity. The third case (a young man 18 years of age,) was chiefly remarkable for the length of time the fetid expectoration lasted, viz. three weeks. In this instance the lower lobe of the left lung was bound down to the chest by pleuritic adhesions, had passed into a state of complete gangrene, and communicated with the upper part of the pleural cavity, which was filled with a sanious fetid fluid, and by which the upper part of the lung was pressed down to the spine. The opposite lung was healthy. Mr. Heaton's case, that of a woman aged 28, is interesting from the circumstances in which it occurred.§ A poisonous dose of opium had induced partial asphyxia, which was followed by subacute and neglected pneumonia. A gangrenous cavity was found after death occupying the greater portion of the upper and middle lobes of the right lung, the walls of which presented a ragged sloughy character, without any evidence of attempt at limitation from the effusion of solid lymph. [This last case, in some measure, confirms the opinion of Chomel, that when gangrene of the lung succeeds to asphyxiating causes, the congestive stage of pneumonia passes at once into gangrene. The age and generally healthy character of the subjects of the above cases are deserving of consideration. In two other cases that will be referred to in a subsequent part of this Report the subjects were young and healthy. A middle aged, athletic looking man was brought into St. Thomas's Hospital, moribund, in August last, who had been suffering under neglected pneumonia for some weeks. The upper portions of both lungs were

* Casper's Wochenschrift, 18 Feb. 1843.
† Gazette Méd. de Paris, March 1843, p. 159.
‡ Report of Cases treated in the Military Hospital at Malta. Edinburgh Med. and Surg. Journal, April 1844.
§ London Medical Gazette, May 3, 1844.

in a state of gray hepatization, and the greater part of the left upper lobe was a mass of gangrene.]

*Cirrhosis of the lung.* Dr. Law* has described two cases of this disease, in which the lung was covered with a dense false membrane, and the parenchyma was also very dense. Drs. Stokes and Greene, have met with two other examples.† Dr. S.'s patient had been labouring, for some months, under cough, with dyspnea and hectic. There was dulness over the upper part of both lungs, without decided signs of cavities. The left lung was the more diseased, and this was found diminished in size, and very irregular on the surface, giving to the hand, when passed over it, the impression of numerous hard bodies, but which were the air-vesicles. In Dr. Greene's case the bronchial tubes were enlarged, and resembled phthisical cavities. The symptoms during life were those, of phthisis.

4. DISEASES OF THE NERVOUS SYSTEM. *Brain.* Dr. John Hughes Bennett‡ has recorded a series of cases of cerebral and spinal disease, with a detail of the morbid changes, as they appeared to the eye with and without the microscope. He distinguishes two kinds of softening, an inflammatory and non-inflammatory, the former being characterized by the presence of exudation-corpuscles and granules, and consisting essentially in the formation and development of nucleated cells, in the liquor sanguinis, effused from the vessels, and acting as a blastema. The non-inflammatory softening he considers to be the result of mechanical disintegration of the nervous tissue, either from maceration in serum, hemorrhagic extravasation, putrefaction, or mechanical violence. It differs from the inflammatory softening in not causing any symptoms during life, being, in fact, a post-mortem change. The two forms cannot, however, with certainty be distinguished by the unaided eye; but the fawn-coloured is generally inflammatory, whilst the red usually depends on congestion or extravasated blood. Purulent infiltration was never found to be a cause of white softening. Contraction of one or more limbs is a common symptom of inflammatory softening. In M. Durand Fardel's treatise on Softening of the Brain§ will be found a very complete history of that disease, a full analysis of which has been given in the 31st No. of this Journal. M. Fardel insists on the importance of distinguishing between the acute and chronic forms, both in respect of the symptoms during life and the morbid anatomy. During its acute stage softening of the brain is specially characterized by redness and diminished consistence, without disorganization of the part, and is by far the most frequent in the convolutions. He contends against the views of Abercrombie and Carswell that the essential character of white softening is a gangrenous condition of the part, arising from ossification of the cerebral arteries, with obstructed circulation. He also opposes the doctrine that this condition of the arteries is the main cause of cerebral hemorrhage in the aged. He considers the white softening of authors simply the chronic stage of red softening, a *colourless* condition consequent on subsequent changes, and the general doctrine which he inculcates is, that softening is an inflammation having nothing special in its nature, essentially the same in the young and the old, whether produced by local injuries, or spontaneously developed. He, however, is compelled to admit a certain number of cases, in which, after an indubitably acute attack, white softening has been detected, i. e. he is compelled to allow the existence of a certain number of cases of *primitive* white softening. [Such cases are most frequent in children. Fardel's observations were made on subjects advanced in life; but it is when occurring in children, that a correct explanation of the nature of primitive white softening, is of most importance. This explanation, however, M. Fardel is not prepared to give.] In a memoir by the same author,

* Dublin Journal of Medical Science, March 1843. † Idem.

‡ Pathological and Histological Researches on Inflammation of the Nervous Centres, by John Hughes Bennett, M.D. Edinb. 1843. Reprinted from the Edinburgh Medical and Surgical Journal.

§ Traité du Ramollissement du Cerveau, par Max. Durand Fardel, M.D. &c. Paris, 1843.

devoted to the investigation of the changes which the blood undergoes when effused in apoplexy, and the consequent alterations in the surrounding textures, will be found a good summary of the morbid anatomy of apoplectic effusions, and their relations to softening of the brain.*

*Brain, abscess of.* A case of encysted abscess of the brain is recorded by Mr. Bolton.† It was of the size of a hen's egg, seated in the posterior part of the right hemisphere, and accompanied by softening which implicated the whole of the posterior and part of the middle lobe. The patient presented during life no symptoms of either muscular or sensorial disturbance. The only symptoms referrible to the head were dullness of manner, dull pain, giddiness and irritability of the stomach. Death occurred in a few hours after the sudden appearance of coma.

*Brain, hypertrophy of.* An instructive case of this rather uncommon affection is recorded by Mr. Wells.‡ A seaman, æt. 24, complained on the 4th of March, of headache, giddiness, and some dyspeptic symptoms, which were relieved by blisters and aperients. On the 27th, he had more giddiness, with general debility. April 1, excruciating pain of the head confined to the occiput, led to the employment of calomel and antimony, and shaving of the head. No constitutional symptoms appeared till the 3d, when there were severe fits of pain, occasional insensibility, contracted pupils, pale face, low pulse, and some muscular rigidity of the lower limbs. The man died on the 7th; occasional fits of insensibility and rigidity of the limbs having recurred at intervals. The dura mater and the arachnoid were found healthy. There was considerable hypertrophy and hardening of the right cerebral hemisphere, which was so much enlarged as to thrust the mesial line to the left of the foramen magnum. Much limpid serum was found in the lateral and third ventricles, and the right hemisphere of the cerebellum was distended by serum. The medullary substance was healthy, and the only other morbid appearance was congestion of both kidneys.

*Scirrhous tumour of the brain, without any corresponding symptoms.* M. Velpeau § relates the case of an old man, æt. 66, who entered the hospital complaining of feebleness, pain between the shoulders, and some incontinence of urine. The pain was relieved by cupping and the incontinence of urine was trifling. His habit of onanism, however, was so inveterate and shameless, that it was requisite to put on a strait-waistcoat. He fell into a state of marasmus and died. No trace of disease of the cerebellum was found, but the whole right anterior lobe of the brain was occupied by a scirrhous mass, which also encroached considerably on the left anterior lobe. A bony scale of some considerable size was attached to the falx. With the exception of a little enlargement of the prostate, all the viscera, &c. were healthy. By the side of these facts, what becomes of all our beautiful physiological theories? asks M. Velpeau.

*Intra-cranial phlebitis.* M. Boudet, of La Charité,|| has recorded the following case. A clerk, æt. 27, of strong constitution, after exposure to wet and cold, was seized with shivering and intense cephalalgia. These symptoms were succeeded by pain in the eyes and photophobia, and eight days after he was bled largely without relief. He subsequently complained of pain in the right shoulder and neck, and the least motion of the head gave him great pain; there was also pain over the orbits and œdema of the right conjunctiva. Slight intermittence of the symptoms led to the employment of quinine, but without

* Archives générale de Médecine, April 1844, Sur la réparation ou cicatrization des foyers hémorrhagiques du cerveau.

† Lancet, vol. i, 1842-3, p. 676. See also Case of Abscess of the Brain, by Dr. G. Pyemont Smith, Lancet, Dec. 31, 1842; and Observation d'Abscès latents du Cerveau, par M. Gouzée, L'Expérience, No. 306, Mai 11, 1843; and Case of Encysted Abscess of the Cerebellum unattended by any diagnostic symptoms, by Dr. Fr. Brown, Provincial Medical and Surgical Journal, Oct. 8, 1842.

‡ Edinburgh Medical and Surgical Journal, April 1844; Report of cases treated in the Malta Naval Hospital.

§ Bullet. Gén. de Thérap. t. xxiv, p. 219.

|| Journal de Connais. Méd. Chirurg. April 1844.

benefit. The pulse became dichrotonous; cupping alone gave any, and that but temporary, relief. Delirium, dilated pupils, with a dry and black tongue preceded death, thirty-one days from the commencement of the illness. In the right middle lateral fossa at the base of the cranium was found a small quantity of yellow viscid pus, without colour; this was within the arachnoid, and covered the inferior portion of the anterior cerebral lobe, the corresponding cortical part of which was slightly softened; with this exception the brain was healthy. The right cavernous sinus was filled with thick glairy pus, and its parietes roughened and unequal; the ophthalmic vein was dilated and filled with pus, and here and there the surrounding cellular tissue was the seat of purulent deposits. The coronary, petrous, and lateral sinuses, as well as the internal and external jugular veins, and the trunk of the brachio-cephalic, and the mastoid and sub-occipital veins were filled with pus. The lungs were the seat of purulent deposits. [Intra-cranial phlebitis appears to have been the origin of the disease, and the pus in the arachnoid had probably escaped from an inappreciable opening.]

*Insanity, Contracted foramen lacerum posterius in.* Dr. Kasloff,* of Kiew, having for some years directed his attention to the state of the great vessels of the head in cases of insanity, has been induced to conclude that, in all its forms, insanity is most intimately connected with derangement of the circulation within the cranium. In particular he has remarked that the foramen lacerum posterius on one, and sometimes on both sides, is much contracted, so as necessarily to have involved great diminution of the jugular vein, and obstruction to the return of blood. The canalis caroticus did not seem to have undergone any corresponding contraction. But along with the contraction of the foramen lacerum posterius was generally seen enlargement of the foramina transmitting veins from the skull. In every skull of maniac or suicide in the museum at Kiew, Dr. Kasloff found this contracted state of the foramen lacerum posterius. [Dr. Kasloff's observations tend to show that a persistent obstacle to the return of blood from the head exists in insanity—an important fact, if true.]

*Parasites.* The zeal with which microscopical investigations have latterly been pursued has led to the detection of numerous parasites, both animal and vegetable, in various parts of the body. It will, however, be impossible to do more than point out the references to some of the discoveries and observations that have been made in this department of pathology. An interesting paper, giving an account of the various entozoa that have been detected in different parts of the eye, is furnished by Messieurs Nordman and P. Rayer.† Filaria oculi humani has been seen in the liquor morgagni, and in the capsule of the crystalline lens; monostomata in the crystalline lens, affected with cataract; distoma in the capsule of the crystalline lens of a child born with lenticular cataract; ecchinococcus between the crystalline lens and choroid; others in the sub-conjunctival tissue, chiefly cysticerci;‡ confervoid growths in the posterior chamber, removed by paracentesis oculi;§ filaria papillosa in the anterior chamber of the eye of a horse.|| Dr. Livois' researches on ecchinococci** led him to examine 800 acephalocysts, and in no one instance did he fail to detect within them ecchinococci. He, therefore, concludes that the true hydatid is nothing more than the containing cyst of these parasites; which, instead of being rare, are among the most common. They occupy different situations in the cyst, according to their degree of development, appearing as granulations or gemmules, adherent to the interior of the cyst as long as their head is not protruded; but when fully developed floating loose in the fluid of the sac. They are generally found in both situations, but sometimes only loose, escaping when the sac is opened. The containing cyst increases as the ecchinococci multiply by reproduction. Prof. Klencke's experiments†† lead him to believe that hydatids may be, and probably

* Zeitsch. für die Gesammt. Med. Jan. 1844.
† Ann. de la Chir. Franc. et Etr. vol. vii, p. 191.
‡ Annales d'Oculistique, March 1842.
§ Casper's Wochensch. Sept. 10, 1842.
|| Oesterreich. Med. Wochens. Jan. 14, 1843.
** Recherches sur les Ecchinocoques. Paris, 1843.
†† Gazette Méd. Dec. 1843.

are, in every case, developed by contagion as he calls it, or by actual introduction within the body. When he injected them within the veins, or introduced them into the cellular tissue or digestive tubes of dogs, cats, birds, &c., he found that they excited the disease. He also detected them in the milk of the cow. Mr. Drewry Ottley has detailed the history of a case in which the ecchinococcus was found in the brain. A new species of intestinal worm has been described by M. Dumeril,* under the designation of opiostoma pontieri. Two examples are recorded of intestinal worms escaping by the umbilicus. One by Dr. Hecking,† in which several lumbrici (spulwürmer) escaped from an abscess over the umbilicus, which closed shortly after. A second by Dr. Siebold,‡ in which a tænia solium escaped from the same situation, and in the same way, without the discharge of fecal matter or gas, or any indication of perforation of the intestine. The fasciola hepatica, so seldom found in the human subject, has been detected in the vena porta by M. Duval.§ The species of demodea (acarus folliculorum) described by MM. Simon and Wilson, has been discovered by Mr. Topping in the pustules of a mangy dog.‖ Vegetable fungi found in the air-sacs of birds, in the mouths of newly-born children, and in the crusta of porrigo favosa, are considered by M. Rayer** as invariably secondary productions. Dr. Remak†† has succeeded in transmitting porrigo favosa by inoculation with the fungi. M. Mandl‡‡ states that the tartar of the teeth consists of the calcareous carapaces of defunct vibriones, which abound in the buccal mucus. Dr. J. Hughes Bennett§§ found, in recently expectorated sputa of a man in the last stage of phthisis, with pneumothorax, cryptogamic vegetation, consisting of jointed, transparent tubes, giving off several branches, mingled with numerous round or oval globules.

### PRACTICAL MEDICINE AND THERAPEUTICS.

### 1. Fevers.

*Influenza.* An epidemic catarrhal fever, presenting all the characters of what is usually termed influenza, has prevailed within the period comprised by this Report, in all the New England States, in which its invasion seems to have been almost simultaneous. The duration of the attack was from a day or two, to a week, or fourteen days; but in the majority of cases a critical sweat, attended by free expectoration, occurred on the third day, with disappearance of the fever on the fifth. Debility and vertigo appear to have been the most constant and remarkable symptoms; but according to Dr. Forry,‖‖ who has given an excellent account of the epidemic, it differed in no respect from others of the same kind. Dr. F. states that in nearly all the cases, at first, a more or less deranged state of the chylopoietic viscera existed. The treatment consisted mainly of mild saline purgatives, diaphoretics and occasionally, when the head was affected, leeches to the schneiderian membrane. Dr. Peebles*** found the eupatorium perfoliatum (vulgo "boneset") very useful in relieving the nervous and muscular symptoms of debility. He ascribes to it diaphoretic, expectorant, and aperient virtues. It was observed by Dr. N. S. Davis,††† that all severe cases were relieved, only after free vomiting, and he was led, therefore, to employ calomel and ipecacuanha, which he found more useful than anything

* Bulletin de l'Acad. Roy. de Méd. 15 et 31 Jan. 1843. † Berlin Medicin. Zeitung, Oct. 19, 1842.
‡ Med. Zeitung von Preuss. No. xvii, 1843. § Gazette des Hôpitaux, Suppl. Dec. 1842.
‖ Physiological Journal, 1844.
** Journal des Conn. Méd. Chirurg. Aug. 1, 1842. See also for other cases and observations on that subject, München All. Zeitung, &c. Dec. 18, 1841; All. Med. Centr. Zeitung, 30 Nov. 1842; and Archives Gén. de Méd. June 1842. †† Berlin Med. Zeitung. Aug. 3.
‡‡ Arch. Gén. de Méd. Sept. 1843. §§ Lancet, June 1, 1844, from Trans. Roy. Soc. Edinb.
‖‖ New York Journ. of Medicin, July 1843, p. 64. *** Amer. Journal of Med. Science, April 1844.
††† New York Journal of Medicine, Nov. 1843.

else, and which never failed to bring away copious dark bilious evacuations, which were followed by speedy relief of all the severe symptoms. The only fatal cases he met with were among those who had been bled. Dr. Gilbert* considered the disease had its seat in the nervous system, but was unconnected with either congestion or inflammation, and was best treated by strychnia.

*Miliary or Sweating Fever.* An epidemic of this disease, so celebrated in former times, but of late rather rare, has, for the space of a year, spread consternation through five provinces of France, la Dordogne, le Lot et Garonne, le Calvados, le Jura, and la Manche. Numerous accounts of the epidemic as it appeared in different parts of France, will be found in the French journals, but the fullest history has been given by M. Borchard in his report to the Royal Society of Medicine of Bordeaux,† and by M. Parrot‡ in a memoir read to the Royal Academy of Paris, on which M. Martin Solon has reported.§ M. Borchard first gives an account of the topography and statistics of La Dordogne, and points out the remarkable fact, that the localities in which marshes and stagnant waters exist, are those in which the disease raged with greatest violence. M. Rayer, in his description of the epidemic of l'Oise in 1821, and in his researches on similar epidemics, has made the same remark. The sweating sickness M. Borchard considers a general disease, depending on a cause which influences immediately and primarily the whole organism, the first shock being felt by the nervous and sanguineous systems, including the blood. All the epidemics of this disease known for a hundred years, have established the fact that the countries invaded have numerous marshes, ponds, rivulets, and forests; and all the historians of the disease have noted the frequent and sudden atmospheric changes, and the disturbances of the natural succession of the seasons. M. Borchard, therefore, concludes that the cause of the disease is an effluvium from stagnant waters, acting under the influence of certain barometric conditions, on localities which, by the vicinity of forests, are sheltered from wind. The epidemic appears to have commenced in La Dordogne in May 1841, and extended south, destroying in Dordogne 797 of 10,805 persons attacked; whilst in the departments of Lot and Garonne only 614 died of 28,307 attacked. Two forms of the disease were generally recognised, a benign and a malignant; but cases which at the onset appeared slight, not unfrequently assumed, suddenly, the malignant form, and proved rapidly fatal. In whatever form it appeared, it was often developed without premonitory symptoms; in other cases severe headache, spontaneous lassitude, and (especially at Perigueux), nausea and vomiting, and pains in the loins announced its approach. Most frequently violent palpitation of the heart and of the cæliac axis, accompanied by severe headaches, attended the onset of the disease; the skin was covered with a general profuse sweat of a very peculiar odour, whilst at the same time, in some instances, it gave to the hand a sensation of burning heat, a sure indication of the nature of the commencing disease. With these symptoms were associated a painful sense of sinking or anxiety at the precordial region, and in some cases violent delirium. About the second or third day, a distressing tingling of the whole surface of the skin occurred, which was followed on the fourth or sixth day by the characteristic eruption. This was, at first, red and apparently papular; but by the microscope could be shown to be vesicular, a character that was discernible the following day by the naked eye. The vesicles, transparent and of the size of millet seeds, appeared at the sides of the neck, on the chest, on the inner surface of the limbs, and sometimes over the whole body. The fluid contained in them had no corrosive property, and when inoculated into other persons produced only a little local irritation. The eruption was discrete, or confluent. On the fourth day it assumed a yellowish tint, and disappeared with more or less desquamation.

* New York Journal of Medicine, Nov. 1843, p. 426. † Gazette des Hôpitaux, 4 Oct. 1842.

‡ Bulletin de l'Académie Roy. de Méd. t. viii, 15 Nov. 1842, p. 105. § Ibid. p. 1018.

Through the entire course of the disease the gastro-intestinal system was free from disturbance, and in some severe cases the appetite was preserved. The complication with nausea and vomiting observed at Perigueux did not occur elsewhere. The tendency to assume a remittent or intermittent type was very generally remarked, though neither the sweating nor the eruption was affected by the exacerbations. The abnormal appearances revealed post-mortem, were various, but were more frequently seen in the brain and digestive organs than in the respiratory, and consisted chiefly of vascular injection and congestion. In some cases nothing was revealed by the examination. The blood obtained by venesection was generally of a bright rose colour, the serum not separating readily, and the clot having usually the appearance of currant jelly, without any buffy coat, except in some few instances. The disorder and desolation caused by the mortality which attended the epidemic led to the trial of various modes of treatment. In some of the more malignant cases the patients sometimes died the third day, bathed in profuse perspiration; but before the appearance of the eruption. In such cases the expectant treatment was soon found insufficient. Antiphlogistic remedies were sometimes useful, in removing local congestions, but failed to overcome the severe symptoms. Purgatives were of no use till about the eighth day, when bilious symptoms showed themselves. In the milder cases, expectant treatment with cooling diluent drinks answered best. But in all severe cases the testimony is almost unanimous in favour of the great importance and utility of quinine or bark. This, in the words of Parrot, "was the anchor of safety." It was given in moderate doses during the short remissions in the early stage of the disease. [The same bad consequences were observed from heaping the patients with clothes and encouraging the sweating, as were noticed by Sydenham and Caius, who also have made the same distinction between the mild and malignant forms of the disease.] An apparently independent epidemic occurred in the east of France in Haute Saone, in March 1842, where it was attributed mainly to the artificial excessively hot temperature maintained by the inhabitants. In Saligny, D. of Jura, the mortality was great, apparently owing to the neglect of quinine in the early part of the epidemic.

*Fever, Epidemic of Scotland.* A very extensive epidemic of fever has prevailed in Scotland, having many of the characters of yellow fever, and which has been described by several very competent observers. In Dr. Cormack's Treatise will be found a full account of the history of the epidemic and its peculiar features; but the distinctive characters are so well exhibited in Professor Alison's short paper, and his high character as a philosophical observer (especially on this particular subject) is such, that his statement will be taken as the basis of the following account of this new epidemic.

After stating* that the ordinary fever had been rare, Dr. Alison says that the great majority of cases are essentially different in their symptoms and progress from strictly typhoid cases, and from any form of continued fever that he has seen generally prevalent: 1. In duration, which is uniformly short; some having a crisis on the fifth, a majority on the seventh, and hardly any protracted beyond the ninth day. When death takes place it is early; in every case he has seen or heard of, before the ninth day. 2. None have shown the true febrile eruption, though some have had petechiæ. 3. An unusually large proportion have become yellow, generally on the fifth day. This has been almost uniformly attended with fulness of the hypochondria, dullness on percussion, and tenderness—generally with much vomiting; sometimes of dark green bile, sometimes of the brownish matter like hare soup, so often seen in cases of organic abdominal disease. He has not seen any black vomiting, but the stools have sometimes had the character of melœna. He thinks the bile, in this state, has never disappeared from the stools, and in fatal cases the bile-ducts were per-

* Scottish and North of England Med. Gazette, Oct. 7, 1843; in London and Edinburgh Medical Journal, March 1844.

vious, and contained (as also the duodenum) an abundance of bile. The liver was enlarged, but not otherwise diseased. Most of the fatal cases have been jaundiced; but many jaundiced patients have done well with little treatment. Most of these have shown the petechial spots, and in one case a blister applied to the epigastrium rose filled with serum quite black. 4. Both in the jaundiced and non-jaundiced cases, there has been much sickness and vomiting, always abating when the critical sweats took place. 5. *Almost every case has relapsed,* the majority on the fourteenth day: the relapses being of shorter duration than the first attack, and seldom attended with jaundice. In all fatal cases there was inflammation to a great extent of the mucous membrane of the bowels. 6. It has abated almost uniformly by critical sweats, preceded frequently by violent rigors. 7. During and after the sweats, there have been severe pains of the limbs, of the character of muscular rheumatism. 8. The mortality has been very small, not more than one in thirty, and chiefly among the old, or where there was obvious complication. 9. *Every pregnant woman has miscarried.* In two the mother died; in one, though the hemorrhage was considerable, the uterus contracted well. In both the fatal cases rapid sinking followed an attack of pain in the side; in one there was pneumonia, and in the other enlarged and softened spleen. The body in this case rapidly putrified, and the blood-globules examined by the microscope, were irregular in form and size. The mode of fatal termination has been like that in other forms of fever, from embarrassment of function and alteration of structure of some particular organ, usually of the brain, with general failure of the circulation.

The great majority of cases required very little treatment. Remedies directed to relieve the symptoms and meet the local complications, generally mitigated the sufferings of the patient, and in some instances diminished or averted the danger. Headache and other uneasy symptoms of the early periods were relieved by bleeding, which, however, neither shortened the disease nor prevented relapses; and some had a slow unsteady convalescence. Leeching, shaving, and sponging the head, with purgatives, gave relief in the early stage, and afterwards an opiate on the third night, and repeated for several nights, was of signal service in palliating the very uneasy sensations of this period. Antimonials and Dover's powder favoured the sweats; leeches, blisters, and calomel and opium relieved the sickness and vomiting. Some with petechiæ recovered rapidly under the use of chlorate of soda: few required large doses of wine.

Does this fever proceed from the same poison as the ordinary typhus, or is it a distinct disease? The one has succeeded the other within narrow limits of time and space in different parts of the town. One man in the infirmary went through both diseases before leaving the ward. The new has differed from other epidemics by the extent and rapidity of its spread in summer time; but in regard to the mode of its extension (by contagion) and the persons liable to be affected, it has differed in nothing from other epidemics. Dr. Henderson remarks,* that a third, fourth, or fifth relapse sometimes occurred, and he met with instances, of the same persons exhibiting the two forms of fever (the epidemic and typhus) within a very short time, which he considers a proof of their distinctness. He noticed also, enlargement of the spleen, and derangement of the functions of the kidneys; and in fatal cases terminating by convulsions, he detected urea in the blood and in the serum, effused within the cranium. [The same condition of the spleen was noticed by Dr. Graves in the Dublin epidemic of some years back.] Dr. Spillan† has called attention to the resemblance of the present epidemic *in all the important features,* to that described by Hippocrates as occurring in the island of Thasus off the coast of Thrace, where the same state of the spleen is referred to. (Vide Clifton's edition of Hippocrates on Air, &c. p. 62.)

In Dr. M'Kenzie's account of the Glasgow epidemic, especial reference is made to the peculiar form of ophthalmia which succeeded to this fever; in describ-

* Edinburgh Medical and Surgical Journal, Jan. 1844.

† London and Edinburgh Monthly Journal, Feb. 1844, p. 176.

ing which he states, that in the greater number of cases all the textures of the eye suffered from inflammation, which therefore, he thinks, may most properly be called *ophthalmitis.* Sometimes, however, the inflammation was confined to one or two textures. It bore most resemblance to rheumatic ophthalmia, or rheumatic iritis; but its closest resemblance was to sympathetic ophthalmia from wounds of the edge of the cornea or sclerotica. His treatment consisted of depletion, mercury, belladonna, and bark.

Dr. Arrott* has described the disease as it occurred in Dundee, where it was preceded by a gradual decline of the ordinary fever. Black vomiting, he states, was common, and the bile generally found viscid and thick. The post-mortem appearances corresponded remarkably with those observed by the French commission in Gibraltar, in 1828, particularly in reference to the condition of the liver, which Louis considers the anatomical character of yellow fever.

*Fever-Typhoid.* M. de Larrogne calls attention† to the admitted occasional absence of all the so-called pathognomonic symptoms of typhoid fever, the rose-coloured lenticular spots, sudamina, diarrhea, pain in the right iliac fossa, meteorism, and nasal hemorrhage, and therefore to their insufficiency as diagnostic signs. From his own clinical observations he believes himself justified in saying, that the four following phenomena are present from the onset, and place the typhoid nature of the fever beyond doubt: 1. Stupor, which presents various shades and forms, according to the causes, peculiarities, and intensity of the disease. 2. Dilatation of the pupils, which is signally invariable. 3. The pulverulence or brownish coating of the interior of the nostrils. 4. Gurgling in the situation of the cæcum, and termination of the ilium, which may be discovered in all cases if sought for with care. Whenever these four symptoms have been noticed, they will certainly be followed by the other phenomena which generally constitute the disease. M. Amedée Latour‡ also, advances the following propositions, though not unreservedly; 1. That the diagnosis of typhoid fever is not so clear, or easy as represented. 2. That some eruptive fevers, variola amongst others, may commence with an assemblage of symptoms identical with those which constitute typhoid fever. 3. That when the varioloid eruption is developed the typhoid symptoms disappear. 4. That these typhoid symptoms coincide with or complicate a number of different diseases. 5. That the typhoid symptoms, in the commencement of these diseases, have no influence on their development or progress. And lastly, that at this period, they afford no indication for special treatment.

Some general remarks on the distinction between typhus exanthematicus and abdominalis, will be found in Dr. Miguel's paper.§ He considers more active treatment to be required in the latter than in the former. Dr. Bartels|| has detailed three cases in which the appearance of sores on the tongue was regarded as critical in the course of typhus fever (*nerven fieber*), no mercury having been given. M. Rayer** has related to the French Academy a well-marked fatal case of typhoid fever occurring in a woman æt. 56, in which the pathognomonic anatomical lesions found after death were equally well marked. A similar case in a woman 63 years of age is given by Dr. Bartlett.†† [In his work on Typhoid Fever, Chomel stated that there was only one authentic case on record of typhoid fever occurring in a person more than fifty years of age; but in 1837, M. Prus read to the Société de Médecine, an example in a woman æt. 78.] Dr. Richter of Dusseldorf,‡‡ relates a case in which *ammonia was excreted by the skin* of a patient suffering under typhus fever. Three days before death, when the patient was in a state of stupor, the face and

* Medical Gazette, 1843, vol. i, p. 225. For further accounts of the epidemic as it appeared in Glasgow, vide Mr. Reid's paper in Lond. Med. Gaz. vol. i, 1843-4, p. 358, and Dr. Smith in Edinb. Med. and Surgical Journal, Jan. 1844.

† Bulletin de l'Acad. Roy. de Méd. t. viii, p. 15.

‡ Bulletin Gén. de Thérap. Dec. 1842.

§ Casper's Wochen. Dec. 17, 1842.

|| Allgem. Med. Centr. Zeitung, 11 and 14 Jan. 1843.

** Bulletin de l'Acad Roy. de Méd. t. viii. p. 37.

†† Boston Med. and Surg. Journ. Oct. 1, 1842.

‡‡ Oesterreich. Med. Wochens. 1843, p. 457.

hair of the head and beard were observed to be covered with a whitish shining matter like spermaceti. On closer examination, the face was found sprinkled with minute spots of a whitish substance, which on being removed, left the skin with a punctated appearance. The excretion continued till death, after which, the thighs also were found covered with small needle-like crystals. Chemical examination proved that the excreted matter was alkaline and ammoniacal, and contained in addition a whitish yellow substance soluble in ether. [Liebig has shown the presence of ammonia in the air in the vicinity of typhus patients; and Donné and Prout ascertained that the rhomboidal prisms in the urine and fæces consist of phosphate of ammonia and magnesia, but the excretion of ammonia by the skin of typhus patients, is a fact equally new and important.] The essays of Mr. Ross, on Typhus Fever, which have appeared in the 'Lancet,' contain the results of his attempts to apply the doctrines of modern organic chemistry to the elucidation of the pathology and treatment of this disease.* M. Rayer's observations can only be referred to as containing much that is interesting in reference to the existence of typhoid fever in animals.†

The following papers elucidating the statistics of fever in Great Britain, are worthy of notice, and may be referred to with advantage. Statistical and Pathological Report of Cases of Fever treated in the Royal Infirmary of Edinburgh, in the year ending September 30th, 1842, by Thomas B. Peacock, M.D.‡ On the Statistics of Fever in St. Thomas's Hospital, with reference to treatment, by H. Burton, M.D.§ This Report contains an elaborate analysis of all the fever cases which fell under the author's care during six years. A Paper on the Statistics of Fever in Edinburgh during a series of nine years, with especial reference to the influence of season, by Dr. Knox.|| The highest average number of cases occurred in the following months, and in the following order: December, November, January, March; and the lowest averages were presented by the months of February, August, and May. Some valuable practical observations on the continued fever of the middle and southern parts of Virginia, from 1816 to 1829, have been given by Dr. J. P. Mettauer.** A systematic Treatise on Fever has been published by Dr. E. Bartlett,†† in which the author endeavours to show that the typhoid fever of France, apparently the most frequent form of fever in America, is a distinct species from the typhus of that country and of England.

*Congestive Fever of America.* Dr. Parry‡‡ has described a congestive fever met with in Central Indiana, of which the following are the chief characteristics. It prevails at the close of summer, and through the autumn, occurring chiefly in the low grounds skirting the rivers. The first symptoms are those of an intermittent; but the first paroxysm is imperfectly marked, and during the interval of twenty-four or forty-eight hours, the patient complains only of malaise. The second paroxysm is very severe, the coldness extreme, and the face and extremities of a death-like hue. In this condition the patient may die or reaetion may take place in three or four hours. During the cold stage of this second paroxysm, there are profuse discharges from the stomach and bowels, either resembling water in which fresh meat has been washed, or containing much blood; intense thirst, extreme restlessness, and desire for air, rapid small pulse, and frequently muscular cramps: but no disturbance of the mental functions, except in the agony of death, or where there is congestion of the head. The usual length of the fatal paroxysm is from three to six hours. The treatment was directed, in the cold stage, to arrest the discharges; for which purpose morphia, camphor, and blue pill, or acetate

* Lancet, Feb. 25 and March 4, 1843.
† L'Expérience, 27th April 1843.
‡ Lond. and Edinb. Monthly Journal, May 1843.
§ London Med. Gazette, vol. i, 1843, pp. 204, 503, 599.
|| Ibid. Aug. 25, 1843.
** American Journal of Med. Sciences, July 1843.
†† History, &c. of Typhoid and Typhus Fever, by Elisha Bartlett, M.D.; Philadelphia, 1842.
‡‡ American Journal of Medical Sciences, July 1843.

of lead, morphia, and calomel were employed. Sinapisms and stimulant frictions to the abdomen were useful, and cupping to relieve local congestion. Internal stimuli were neither productive of good nor harm; but iced drinks were of use. If reaction could be established, quinine became *the* remedy, and rarely failed to save the patient.

*Erysipelatous Fever.** A malignant form of fever attended at the onset with sore throat, and subsequently with erysipelatous inflammation of the integuments, and often of the internal serous membranes, was epidemic in Vermont and New Hampshire, in 1842-3. The inflammation of the skin and cellular membrane was such as to produce disorganization and separation of contiguous parts to a great extent. A semi-putrid thin fluid was infiltrated into the cellular tissue, so acrid that when discharged the "*hardest steel was directly penetrated by it, as by nitric acid,*" and instruments used to open abscesses were, after a few hours, "*entirely eaten through.*"!! When internal organs were affected, the patients almost invariably died. During the prevalence of the epidemic, cases of puerperal peritonitis, of the same character, were sensibly increased, and in many instances appeared to be communicated by the medical attendant.

[The appearances in the abdomen after death, both of non-puerperal and puerperal cases, were precisely those that have so often been described as marking the true malignant puerperal fever of this country.]

An account of the same, or a similar epidemic, occurring in Riply and Deartown counties, Indiana, is given by Dr. Sutton,† who also subscribes to its contagiousness. Dr. Allen, in his account of the same disease, states that "when the manifestations were external, and the inflammation of the skin did not recede, there was little or no danger to be apprehended;" "but that if the local affection manifested itself either primarily, or by metastasis, in a vital or internal organ, the most serious consequences were to be apprehended. The local manifestations were often primarily denoted by an internal organ. The true character of the disease could then only be ascertained by the severity of the chill, ensuing heat, and the kind of diseases prevailing."‡

*Intermittent Fever, condition of the Spleen in.* M. Piorry, in a memoir presented to the Academy of Sciences,§ has given the conclusions to which he has been led by the consideration of 165 recorded cases, and upwards of 1000 others of which he has no written record. The conditon of the spleen was ascertained by means of the plessimeter and percussion, and the results are, therefore, in the author's estimation, of the utmost certainty. From the analysis of 163 cases, he considers it certain that ague occurs, independent of miasmatic causes; and that in many instances it arises from falls, blows, and inflammation of the spleen. Enlargement of this organ is so frequent in ague that, in 154 of 161 cases, it exceeded the normal size; and in four of the remaining seven it was painful, which was also the case in eighty-two of the whole number. Splenic pains sometimes precede the fever. Organic affections of this viscus may either produce or keep up intermittents. He thinks there is no evidence that any persistent alteration of the blood can directly produce ague. Miasmatic causes act through the nervous system of the spleen. Sulphate of quinine quickly dissipates a large majority of the cases of enlarged spleen, and even in its healthy state its volume may be reduced by the introduction of quinine through the stomach or bowels. In two cases he thinks fatal hemorrhage might be attributed to the too rapid diminution of the spleen under the influence of quinine; hence the dose of this medicine should be proportioned to the enlargement. The quinine, he believes, is absorbed by the veins, and cures ague by its direct action on the spleen. A case of intermittent fever observing a septan period, is recorded by Dr. Laroche (père) of Angers.||

* American Journal of Med. Sciences, Jan. 1844, p. 13. Account of the erysipelatous fever, as it appeared in the Northern section of Vermont and New Hampshire, in 1842-3, by Drs. Hall and Dexter.

† Ibid. same date, p. 247.

‡ Boston Med. and Surg. Journal, vol. xxx, No. 2, et seq.

§ L'Examinateur Médicale, No. 13, t. iii.

|| Archives Générales de Méd.; Mars 1843.

*Intermittent Fever, Treatment of.* Dr. Cenni,* in a letter to the Editor of the Racogliatore Medico, states that many cases of ague, resisting all other plans of treatment, he has found give way to mercury associated with quinine. Tenderness of the spinal column, in persons affected with intermittent fever of a chronic form [previously noticed by MM. Griffith and Van Mons], M. Gouzee† has found to occupy chiefly that portion included between the third and fifth dorsal vertebræ. In such cases he has derived from local depletion and counter-irritation great success.

M. Chomel‡ has given an abstract of his experiments with various so-called febrifuges in the treatment of intermittent fever, (which illustrate in a striking manner, the fallacies to which we are exposed in estimating the value of new remedies.) Powdered holly (" poudre de houx") having been much vaunted, he proposed to treat with this substance twenty-two patients, but seven had no paroxysm, after admission into the hospital (La Charité); of the remaining fifteen, eight had other slight diseases, such as bronchitis, and were cured without the aid of any specific febrifuge. In four of the remaining seven, the paroxysms became daily more slight without the interference of medicine, and in the other three the paroxysms returned with regularity, and were of an unyielding and fixed character. In these the powdered holly was tried and given to the extent of ℥ij for a dose, without any benefit; but on exhibiting quinine speedy cures were obtained. Had the holly been given to the whole twenty-two, it might have had the credit of curing nineteen, and failing only in three, a conclusion manifestly false. He tried salicine in the same way, and found it equally inefficacious, and the whole of his observations have led him to conclude that cinchona bark is the only febrifuge.

### 2. Diseases of the Digestive System.

*Stomach.* After noticing that the act of regurgitation is often a normal one, even in man, being the natural means of relieving an overloaded stomach; that it is seen in infants, who by what is called "posseting," expel the superabundant portion of milk from the stomach; that at all ages the stomach is thus enabled to expel gases or the morbid fluid secretions which characterize certain forms of dyspepsia; and that some persons possess the power of regurgitating their solid food, remasticate it, and again swallow it like ruminating animals; Sir H. Marsh§ proceeds to point out that in other instances, regurgitation of food without nausea constitutes a distinct and peculiar disease. Many of the cases observed by the author were evidently of hysterical origin.

The second case given in illustration, is that of a young woman, æt. 26, who previously to being affected with this stomach affection, had suffered from almost every possible variety of hysteria, and among others, from distressing and obstinate cough occurring abruptly, at the same hour each day, for many months. Some time after this, having been in good health for several months, she was seized with nausea and vomiting to such an extent that scarcely any food remained on her stomach. The nausea ceased in about three weeks, and the food instead of being vomited was regurgitated, and few articles would remain on the stomach. This regurgitating action of the stomach had a periodic character. It was preceded by an oppressive sense of fulness and weight at the epigastrium, which was removed by the ejection of the food. The spine was occasionally, and at various points, slightly tender on pressure. A variety of remedial means were used, and in some instances, with striking though temporary benefit. The last remedies employed were iced coffee internally, and crude ice externally to the epigastrium. In another instance, violent epileptic paroxysms occurring every hour for twenty-four hours, were succeeded, first by diarrhœa, and then by regurgitation of food; and the author has seen several other well-

* Gaz. Medica di Milano, Sept. 16, 1843. † Annales de la Soc. de Méd. d'Anvers, March 1843.
‡ Discours d'Ouverture, &c.; Gazette des Hôpitaux, 19 Nov. 1842.
§ Dublin Journal of Med. Sciences, July 1843, p. 437.

marked instances of the same disease in young children, resulting from innate constitutional delicacy, and coexisting with various disturbances of the nervous system, and of the digestive functions. He has seen instances of it in conjunction with chorea, and remarks that in many cases of hooping-cough, the partial or total evacuation of the stomach, at the close of the fit, is the result, not of the act of vomiting, but of regurgitation. In another class of cases, this peculiar disease holds a prominent position among many symptoms which appertain to obstinate and protracted forms of dyspepsia. Illustrative cases are given.

A case of *pyrosis* occurring periodically in a young girl æt. 12, is recorded by Dr. Eitner.* The fluid was discharged with great force by sudden vomiting, and in enormous quantities. It appeared to collect gradually in the stomach, when pain and oppression of the epigastrium occurred, which were relieved by vomiting. The disease gave way speedily to a mixture containing extract of quassia, with laurel-water and carbonate of potash; a camphorated ointment with laudanum being rubbed over the stomach.

A fatal case of *hæmatemesis* occurring, without any assignable cause in a previously healthy man, is recorded by Dr. Laroche.† The patient, a soldier, who had never been known to complain of any dyspeptic symptoms, was suddenly seized with profuse hemorrhage from the stomach, of pure, florid blood, and speedily died. The stomach was found filled with black blood, but though carefully examined, presented no lesion, except a little softening of the mucous membrane. All the other organs were sound.

Dr. Imray‡ has given an account of the "*mal d'estomac or cachexia Africana*," as seen among the negroes of Dominica; from which it appears that there is nothing peculiar in the pathology of the affection. The chief characteristics seem to be derangements of the functions of digestion and assimilation. The mucous membrane of the stomach and intestines was sometimes merely bloodless, at other times it was softened, whilst occasionally there was ulceration with scirrhous thickening near the pylorus; the mesentric glands were frequently enlarged and "diseased." Dr. Imray's treatment consists in the exhibition of ferruginous tonics, combined with antacids.

*Dysentery* has been epidemic in various districts of France during the period comprised by this Report. M. Peysson§ (military surgeon at Lyons) is convinced of the superiority of venesection over every other mode of treatment. He employed it successfully in 1840, in more than 300 cases, but no mention is made of the number of fatal cases. M. Senac, of Lyons, bears testimony to the success of this mode of treatment.

M. Labarthe ("aide major" of M. Peysson)|| details with the utmost brevity, eighty-six cases occurring under the care of M. Peysson. Seventy-three of these are given in about the same number of words, eight are detailed more fully, but very imperfectly. Fourteen cases were admitted into hospital in the month of June, twenty-three in July, and forty-four in August. The mean time that the disease had existed before admission into the hospital was seven days. All were soldiers. The treatment consisted principally of general bleeding, cupping, leeches, mucilaginous drinks, and mixtures, frequently containing opiates, emollient lavements, and poultices to the abdomen. The quantity of blood drawn, at first, was, in thirty-five cases, 20 oz.; in twelve, 18 oz.; in nine, 16 oz.; in nine, 15 oz.; and in sixteen, the quantity is not mentioned. A second bleeding was adopted in twenty-three cases, and a third in three cases. Opiates were administered to sixteen or seventeen. The average time passed in the hospital was seventeen days. Three patients died, or 1 in 25; and five re-

* Medicin. Zeitung, Feb. 22, 1843. † Archives Gén. de Méd. Fev. 1, Sec. 4, p. 362.
‡ Edinburgh Medical and Surgical Journal, No. 155.
§ Gazette des Hôpitaux, Sept. 20, 1842; from Bull. de la Soc. de Méd. de Toulouse.
|| Bulletin de l'Acad. Roy. de Méd. 15 et 30 April 1843.

quired leave of absence from the army for convalescence. [This treatment, by no means new, was practised by Cleghorn at Minorca, in 1744-49; and by Lamoricière at Lyons, in 1625, as well as by others.] The epidemic prevailed in many of the communes of the Loire Inférieure, and raged with great intensity at Ebray, near Chateaubriant, where many died. M. Aubrée* treated twenty-seven cases and cured twenty-five. His treatment consisted in the administration of an emetic, and subsequently of an infusion of bark with gummy extract of opium, three times a day, and the following pills: ℞. camphoræ, 23 centigr.; extr. opii gumm. 15 centigr.; extr. cinchonæ, 2 grammes; M.f. pil. viij: S. j. horâ somni. The patients had a tisan of althæa (palustris?), for drink, emollient poultices to the abdomen, and lavements of linseed and mallow were used daily. M. Aubrée ascribes the epidemic to ergot of rye, more or less of which he states entered into the composition of the rye bread of the peasants.

In the epidemic as it prevailed near Bordeaux in 1842, M. Chaberly† tried the antidysenteric emulsion of Bouchardat (Annuaire de Thérapeutique, 1841,) which consists of a decoction of Iceland moss made into an emulsion with poppy seeds, to which are added syrup of poppies and syrup of quinces. From this, preceded by antiphlogistic treatment, he derived good results. But its dearness led him to try a decoction of acorns, previously slightly roasted. This he found exceedingly useful in both the acute and chronic forms of the disease, especially in children. MM. Masselot and Follet,‡ in a lengthy memoir, have described the epidemic as it reigned during the months of August, September, and October, 1842, at Versailles. They came to the following among other conclusions: That the disease is not an enterocolitis, but a general affection of the whole system, depending on a cause allied to marsh miasm. The intestinal ulceration which is always present, after the sixth day, is not due to inflammation, but to softening of the mucous membrane, or gangrene. False membranes in the alimentary canal are never met with in the acute stage. The treatment has yet to be sought. An epidemic presenting an adynamic character, prevailed in the autumn of 1841, in the district of Salzburg, and has been described by Dr. Wittmann of Radstadt.§ Its duration was two and a half months, and the number attacked 263, of whom 203 recovered and 60 died. In one case dropsy of the cerebral membranes occurred and gave rise to severe tetanic symptoms. Post-mortem examinations revealed inflammation and ulceration from the rectum through the colon and cæcum and even into the small intestines. The treatment consisted of tonics, especially the stimulant, emetics, alteratives, and counter-irritants, but neither mercury nor venesection was employed.

*Peritonitis, Strumous.* Sir H. Marsh, Bart., and Dr. F. Churchill,‖ have detailed several cases of strumous peritonitis, with effusion, and given some valuable remarks on the diagnosis and treatment of the affection, which, they observe, sometimes assumes an acute character, but is more frequently met with in the chronic form, in which the early symptoms are very obscure. It is said [contrary we believe to the generally received opinion] oftentimes to yield to judicious treatment, if early detected. It is confined to persons of from three or four to about thirty years of age. In many cases the fluid is limpid and serous, and may be wholly absorbed, and leave but few adhesions. The gradually increasing distension of the belly has, in more than one instance, been mistaken for pregnancy. [Their observations do not confirm Louis' statement, that when occurring in adults, the lungs always present tubercles. He affirms that the existence of tubercular peritonitis alone will justify the diagnosis of pulmonary tubercles, though no pulmonary symptoms may be present.] In two of the cases detailed by Sir H. Marsh, occurring in adults, it is distinctly stated that the lungs were free from all traces of tubercle. Attention to the history of the case, and to the early symptoms, affords the chief means of diagnosis.

* Gazette des Hôpitaux, Nov. 1, 1842.

† Bulletin Gén. de Thérap. 15 et 30 April, 1843.

‡ Archives Gén. de Méd. p. 147, et ante 1843.

§ Med. Jahrbuch. d. Oester. Staats. Nov. 1842.

‖ Dublin Journal of Medical Science, 1 March, 1843.

Diarrhœa is a very frequent accompaniment, at first often the main symptom, and should not be suddenly checked by astringents, but is best treated by leeches, blisters, fomentations, and anodyne enemata. The remedies of most importance are topical bloodletting, blisters, diuretics, mercury, and iodine. When the most acute symptoms have thus been subdued, diuretics, particularly inf. digitalis, with nitrate of potash, are of great use. "But of all the curative agents we possess, that which is most valuable is mercury; of all diuretics it is the best: in some cases it must be resorted to at once, in others it is necessary as a preparatory step, to subdue the more acute symptoms by detractions of blood." It is best introduced by inunction, and is sometimes with advantage conjoined with iodine. Iodine internally is occasionally useful, particularly when it acts as a diuretic; and for this purpose it should be associated with liq. potassæ. Sir Henry thinks the opinion that mercury is inadmissible in strumous disease in general, not well founded. Dr. Churchill confirms the utility of the treatment recommended by Sir H. Marsh; [and his statements respecting the symptoms and diagnosis correspond with Abercrombie's.] "In those cases where there is no pain and but slight tenderness, with little disorder of the digestive organs, the principal guide to diagnosis is the enlargement of the abdomen, which ultimately always occurs, and the fluctuation which, by a little care, may always be perceived." A case recorded by Dr. O. B. Bellingham* offers a good illustration of the obscurity of the symptoms. The patient, a woman æt. 26, presented as the only constant symptoms, a tumid state of the abdomen, quick pulse, emaciation, and a dry but not coated tongue, redder than natural at the tip. The case proved fatal by perforation of the walls of the intestine. Numerous tubercles, and some lymph were found on the peritoneum, but *the lungs were quite healthy.* Dr. Meredith Clymer† contends that the evidence at present collected, does not justify the conclusion to which Sir H. Marsh has arrived, that mercury is the chief remedy. In a paper by Dr. A. Toulmouche of Rennes, will be found some further observations on this subject, especially with reference to the causes of the difficulty of diagnosis.‡

*Ascites.* In M. Velpeau's researches§ on the physiology and pathology of the shut cavities of the body, will be found some experiments on the injection of dilute solutions of iodine into the abdomen, with a view to determine how far this procedure might be adopted for the radical cure of ascites. A remarkable case of ascites is related by M. Cann of Yvetot.|| The patient, a female servant, when 36 years of age, had an attack of entero-peritonitis, which passed into a chronic state, and three months after, the urinary secretion became suppressed, and ascites supervened. From this time, it became necessary to tap her occasionally, to relieve the extreme oppression of breathing. The peritoneum after the operation could be felt to be uneven, tuberculated, and hard. After tapping his patient every six, eight, ten, or twelve days, during a period of fifteen years, M. Cann determined to try pressure by means of card-board passed round the abdomen between folds of linen. This the woman could not endure for more than three days, but from this time the urine became more abundant, and the fluid in the abdomen accumulated less rapidly, so that the intervals between the operations were gradually extended; the last amounting to six months. From this time the patient continued well, gaining flesh and strength. Some enlargement of the abdomen, however, remained, and glandular (fleshy) masses could be felt over different parts, especially over the colon. During a period of sixteen years, the patient was tapped 886 times, and a quantity of fluid was removed, estimated at the enormous quantity of 173 hectolitres and 30 litres, [or upwards of 3812 gallons, imperial measure ! !]** Professor Gintrac†† has de-

* Dublin Medical Press, July 5, 1843.

† Philadelphia Med. Examiner, Nov. 11, 1843.

‡ Gazette Médicale de Paris, Nos. 35 and 49, 1842.

§ Annales de Chirurgie, April and May, 1843.

|| Bulletin de l'Acad. Roy. de Méd. t. viii, p. 77.

** See also a fatal case of enormous hydropic distension of the abdomen, which contained fifty-nine pints fluid imperial measure, by Sir D. Dickson; Edinb. Med. and Surg. Journal, Jan. 1843.

†† Journal de Médecine de Bordeaux, Jan. 1844.

tailed a case of ascites [presenting many points of interest] produced by ossification and obliteration of the vena portæ. All the abdominal veins which ended in the splenic and superior mesenteric veins were gorged with blood. The liver was pale and irregularly wrinkled or mammillated, and the gall-bladder contained a moderate quantity of thickish bile. M. Gintrac concludes that though the obliteration of the vena portæ modifies, it does not prevent, the secretion of bile; though it materially interferes with the nutrition of the liver. Dr. Debavay* relates a case of peritoneal dropsy succeeding to puerperal peritonitis, and treated with various remedies with but temporary relief. The abdomen remained tense, hard, and presented a distinct sense of fluctuation. The abdominal organs appeared sound, and the general health was good. Dr. Debavay, determining to try the effect of arsenic, gave $\frac{1}{20}$ of a grain night and morning, in the form of pill. After some days, when three pills a day were taken, it was necessary to suspend them in consequence of colic pains and diarrhœa. They were, however, again resumed, one or two being taken per diem. At the end of six weeks, the abdomen was less tense, and diminished in size; the urine more limpid, and increased in quantity. The skin, before dry, became soft and moist. The arsenic was continued, at intervals, for six months, when all symptoms of dropsy had disappeared.

*Constipation and Ileus.* Several remarkable cases of protracted constipation have been recorded. One related by Mr. Chalmers, of the Cape of Good Hope,† occurred in the person of a young woman æt. 20, who for three years was unable to empty the bowels without artificial aid; and indeed from her birth required the introduction of a piece of soap, or some other means to procure an evacuation. For three years the appetite was so impaired that all were astonished that life could be supported with so little food. There appeared to be some obstruction in the sigmoid flexure of the colon; but fæces in the rectum were never discharged without some artificial aid. During four months only two evacuations were obtained. At the date of the last report, the constitutional powers were beginning to give way. Dr. J. Johnson‡ related to the Westminster Medical Society an instance in which the constipation, induced by scirrhus of the rectum, lasted forty-five days. Dr. Burne's tube could not be introduced, and all medicines failed to produce any effect. All ingesta were vomited, but there was no stercoraceous vomiting. The constipation set in suddenly. Half a pound of fluid mercury which had been given five days before death, could not afterwards be discovered in the intestines. A similar case is recorded by Mr. Wallis, of Castle Cary,§ in which the constipation lasted forty-three days.

Three cases of *obstructed intestine and recovery after fecal vomiting* are recorded: one by Sir George Lefevre,‖ in a girl æt. 11, who recovered after the ninth day of obstruction, by the spontaneous evacuation of the bowels, attended by much vomiting of dark green matter; and a second by Dr. Mayo,** in an old lady, who recovered after fecal vomiting of seven days, on passing a large intestinal calculus. A third case is related by Dr. Staal,†† in which, after the occurrence of stercoraceous vomiting, the patient was completely relieved by the injection of gr. iv. extr. belladonnæ in gruel. Narcotic symptoms ensued, with speedy fecal discharge, and the patient was well in two days. Dr. Kosching relates a case of *ileus*, occurring to a man whilst loading a waggon, in the course of which he had frequently to mount and jump down. The lower extremity of the ileum was found simply twisted once, on itself, and when untwisted was quite pervious. The surrounding intestines were much distended with gas, and slightly reddened. [Would not crude mercury probably have saved this man?] M. Heldenbergh‡‡ states that in a case of ileus, in which constipation had lasted six days, and the symptoms had become threatening, evacuations, and complete relief were obtained by five-grain doses of sulphate of quinine, repeated

* Gazette Méd. de Paris, 9th March, 1844. † Med. Gazette, Sept. 30, 1842.
‡ Lancet, Nov. 26, 1842. § Ib. Dec. 17, 1842. ‖ Med. Gazette, vol. ii, 1843-4, p. 26.
** Med. Gaz. May 3, 1844. †† Oppenheim's Zeitschrift, Feb. 1844, p. 258.
‡‡ L'Expérience, March 21, 1844; from Bull. de la Soc. de Méd. de Gand.

every half hour, for thrice. The constipation returning, was again relieved by quinine.

*Tympanitis.* In a case in which intestinal disorder was attended by great tympanitic distension of the bowels, M. Levrat (ainé de Lyon)* had recourse to paracentesis of the small intestine over the most salient point. The operation was per formed with a trochar of the size of a stocking-needle [apparently similar to Dr. Babington's,] and gave immediate relief. Fifteen days afterwards the patient was about his business. In the case already referred to of so-called gangrene of the lung, terminating by perforation of the diaphragm and peritonitis, which was attended by great tympanitic distension of the abdomen, the peritoneum was four times punctured with considerable temporary relief.†

*Recovery after perforation of the ileum in fever.* In a case of fever, occurring under Louis'‡ care in the hospital Beaujon all the symptoms of perforation in the vicinity of the ileum occurred on the 10th or 12th day of convalescence. The patient recovered after [certainly not in consequence of the] treatment, which consisted of *enemata,* leeches to the abdomen, and three small doses of morphia.

*Liver, fatal spontaneous hemorrhage from.* Dr. Levieux (fils)§ in a memoir on lesions of the abdominal organs, considered as the causes of sudden death, has related a remarkable example of sudden death from hemorrhage from the liver without effusion into the abdominal cavity. A custom-house officer, aged 43, having always enjoyed and being, at the time, apparently, in perfect health, after having been actively employed all day, dined with his fellow-officers, and dropped suddenly dead. Nothing in the head or chest could be detected to account for death. The abdominal organs were, however, all gorged with blood, and the liver enlarged, but its external aspect natural. Internally, it presented all the characters of incipient putrefaction, both as regards colour and density, was readily broken by the finger, and gave exit on each incision by the scalpel to a large quantity of blood. The gall-bladder, duodenum, and small intestines were filled with blood of the aspect of wine lees. The stomach contained a large quantity of food mixed with a blackish liquid, of a nauseous smell. The large intestine was distended with gas. No ulceration or rupture of any part was detected. M. Levieux considers the case as a species of apoplexy of the liver, the blood having been discharged by the biliary ducts into the duodenum, &c., without rupture of the liver. The following case is recorded by Dr. James Abercrombie.‖ A lady who, during her pregnancy, had suffered from dyspeptic symptoms, was suddenly seized, soon after delivery, with pain in the right hypochondrium, followed by symptoms of collapse, threatening immediate death. There was no uterine hemorrhage, nor could any satisfactory cause be assigned for the symptoms, which gradually subsided, so far that she lived till the following day, when an attack of shivering supervened, and was followed by vomiting, and all the symptoms of collapse of the previous day. In twelve hours after she died. On laying open the abdomen, a large sac presented itself, occupying the superior and anterior surfaces of the liver. On attempting to remove this organ, about lb.ij. of fluid and coagulated blood escaped, and two small openings, about an inch apart, were detected in the liver, through which the blood had escaped from a branch of the vena portæ. The sac proved to be the peritoneal covering, which had been detached by the effused blood. The liver was throughout diseased, mottled, and exceedingly friable. The uterus and all the other viscera were perfectly sound.

*Liver, tumour connected with.* Mr. Barlow, of Writtel,** Essex, relates a case in which, after injury, the patient suffered from pain in the region of the liver, with symptoms of collapse. Two days after, the motions were white and the

* Bulletin de l'Acad. Roy. de Méd. t. ix, p. 9. † Journal des Connais. Med.-Chirurg. Nov. 1, 1842.
‡ Archives Gén. de Méd. p. 74, 1843.
§ Annales de la Chirurgie, Fr. et Etr., in L'Expérience, April 25, 1844.
‖ Medical Gazette, Sept. 13, 1844. ** Ib. May 24, 1844; Roy. Med. et Chir. Trans.

urine dark, as in jaundice. A swelling gradually appeared over the region of the liver, which at length, from its size, produced so much distress, that it was punctured, and seven quarts of fluid discharged with great relief. The fluid had all the appearance of pure bile, and the analysis of subsequent tappings proved it to be so. The man was tapped four different times subsequently; on the last occasion the tumour was not emptied, and pain was felt; but on the following day bile appeared in the stools, the urine became paler, the swelling gradually subsided, and the patient recovered. [? Distended gall-bladder, or effusion of bile behind the peritoneum, from rupture of the liver.] An example of fatty liver, unconnected with scrofula, is related by Dr. Watson in a clinical lecture.*

*Biliary calculi, jaundice, &c.* M. Duparcque† considers clonic spasms of the right hypochondrium, extending down the side and followed by convulsive movements of the muscles, pathognomonic, of impacted biliary calculi. In four cases out of thirteen in which retention of bile was produced by this cause, the above symptom was present, and he has met with it in no other disease. His remedy (Durand's) is a mixture of ol. ricini and æther. In a case of jaundice related by Dr. Graves,‡ the only lesion discovered after death was inflammation of the mucous membrane of the gall-bladder. The attack commenced with pain in the right hypochondrium, extending towards the epigastrium, to which succeeded the usual symptoms of icterus. The pain was persistent, and the patient saw everything of a yellow colour. Death was preceded by delirium and coma. The lining membrane of the gall-bladder was the seat of intense inflammation, and covered with coagulable lymph, but the inflammation did not extend to the ducts. Dr. Seeger§ reports the case of a woman previously healthy, who after suffering from colicky pains and tenderness of the right hypochondrium, presented a tumour at the umbilicus, from which were discharged for a length of time a number of gall-stones.

*Pancreas.* Dr. Claessen's treatise on diseases of the pancreas|| contains a vast amount of materials for the elucidation of diseases of that organ. In reference to diagnosis, he states that though in thirty cases there was a watery discharge from the mouth, he objects to the inference that this intimates either increased pancreatic secretion or vicarious action of the salivary glands. He rather refers it to the stomach, more particularly as the pyrosis was frequently associated with vomiting and other evidence of gastric disturbance. He therefore, places no confidence in the diagnostic value of pyrosis. Pain and costiveness are frequent symptoms of pancreatic inflammation. Dr. Batersby** has also collected a great deal of valuable information on the obscure subject of pancreatic disease. In one of the cases detailed by himself the diseased pancreas was at first mistaken for aneurism of the aorta; and in a second case, disease of the pancreas was diagnosticated by some German physicians attending Dr. Graves's clinique, from the extreme moisture, cleanness, and macerated appearance of the tongue and mouth generally. In the former cases the same state of tongue existed, and there was also salivation. Dr. B. alludes particularly to the diagnostic importance of both salivation and pyrosis, and of the sympathy existing between the buccal and abdominal salivary glands.

*Spleen, removal of.* M. Berthet de Gray†† relates the following case. A middle-aged man received a wound in the side, through which the spleen eventually protruded, and becoming gangrenous, was removed. The man recovered and lived thirteen years, enjoying sound health, his digestion being usually good. After death, produced by pneumonia, all that remained of the spleen was found to be a small portion of the size of a filbert, adhering to the stomach. Mr. Eagle‡‡

* Provincial Medical and Surgical Journal, Nov. 4, p. 106. † Revue Médic. April 1844, p. 506.
‡ Dublin Journal of Med. Science, Nov. 1843. § Oesterreich. Med. Wochens. Feb. 4, 1843.
|| Die Krankheit. d. Bauchspiecheldrüsen, van D. H. Claessen; Koln, 1842.
** Dublin Journ. of Med. Sciences, May 1844.
†† Scéance de l'Académie Royale de Médecine, 9 Juillet, 1844. ‡‡ Lancet, Oct. 8, 1842.

asserts that fattening and cicatrization of the tubercles were the results of the removal of the spleen in his experiments on rabbits affected with tubercle (?) of the liver and marasmus; he therefore proposes to tie the splenic artery in patients moribund from inanition, arising from disease of the nutrient circulation, rather than from structural disorganization, as in some cases of phthisis and marasmus!!

### 3. Diseases of the Respiratory System.

*Bronchitis, &c.* An epidemic catarrh prevailed at Nantes in 1840-41, of which an account, as it was observed in the Hôtel-Dieu of that city, has been given by M. Mahot and his colleagues.* It attacked principally the soldiers of the garrison, and especially the young recruits. It appeared under two forms: one simple, analogous to the influenza—the other a "suffocative capillary bronchitis," a very severe disease, having the following characters: 1, succeeding to an attack of acute catarrh; 2, expectoration of thick yellow sputa; 3, extreme acceleration of the pulse; 4, death supervening suddenly on any movement of body; 5, softness of the substance of the lungs, and an abundance of whitish, or yellowish muco-purulent matter in all the bronchi, sometimes with lobular, or simple pneumonia. An account of a similar epidemic, which prevailed in Paris in the spring of 1840, has been given by H. Lasserre.† It was observed by him in La Pitié hospital under Piorry. Of thirty-one cases, two terminated fatally. Tartar emetic was the medicine chiefly relied on. From the thesis of M. Foucart,‡ the disease appears to have consisted in an acute inflammation of the smaller bronchi, going on to purulent secretion, but not producing hepatization, and to have corresponded closely with the "catarrhe suffocante" of Laennec. A well-marked case of *hay asthma* is recorded by Mr. Cheyne,§ in which the wife of a stable-keeper (whose lofts were filled with hay just brought in, and having an unusually powerful odour) received no relief from ordinary remedies, but who was speedily relieved of all the distressing symptoms on removing to lodgings, only one hundred yards distant. M. Gerard|| calls for farther attention to the efficacy of *emetics in the early stage of acute bronchitis.* In several cases that he adduces, in which the pulse was upwards of 100, and considerable pyrexia was present, all the febrile symptoms rapidly subsided, after two or three emetics of ipecacuanha and tartar emetic.

*Foreign bodies in the bronchi.* The interest excited by the case of Mr. Brunel has brought to light many curious facts relating to the introduction of foreign bodies into the bronchi, and given rise to much discussion on the treatment of such cases. Mr. Brunel's case has been detailed by his medical attendant, Sir B. Brodie, before the Medic. Chir. Society.** On the 3d of April, while playing with a half-sovereign, the coin slipped behind the tongue, and passed into the larynx. This was followed by a fit of coughing, severe sense of choking, and vomiting. On the 6th and 7th the cough returned, and after exposure to cold, became worse, and was attended by the expectoration of mucus and a little blood. Pain in the right side of the chest was now complained of. On placing himself in an inverted position over the back of a chair, a loose substance was felt to be dislodged, and passed up to the larynx, when the pain in the chest was relieved. Nothing abnormal could be detected by the stethoscope. On subsequently inverting the body (by means of a machine on which the patient was laid, and by which either the head or feet could be elevated at pleasure,) and striking the back, the cough suddenly became so violent and the sense of choking so severe, that it was deemed prudent to desist. On the 27th tracheotomy was performed, and on two occasions it was attempted to remove the coin by the forceps, but the convulsive coughing thus excited rendered the attempt vain. On the

* Relation d'une Epidémique de Bronchite, &c., Nantes, 1842.; reviewed in Bull. Gén. de Thérap. 15 et 30 Oct. 1842.
† Archives Gén. de Méd. Oct. 1842.
‡ Gazette des Hôpitaux, Oct. 27, 1842.
§ Med. Gazette, Dec. 2, 1842.
|| Arch. Gén. de Méd. t. iii, 1843, p. 195.
** Lancet, vol. iv, 1842-3, p. 480.

13th of May the aperture in the trachea having been kept open, the body was again inverted on the plane, and the back being struck two or three times, the coin passed into the mouth, and fell against the incisor teeth; a little blood was passed at the time, but there was no cough nor any convulsive distress, such as had before occurred. From this time he rapidly recovered. The following cases may also be referred to. A case in which tracheotomy was performed, and a stone was removed from the right bronchus of a child æt. 6, by means of slightly curved polypus forceps. No respiration could be heard over the right lung previous to the operation.* A case in which a beech mast was retained in the air-passages for nine years and a half, and the patient, a girl æt. 16, recovered after its spontaneous ejection. The symptoms had been cough and periodical discharge of pus, but none of the constitutional symptoms of phthisis. The growth and development of the frame had, however, been arrested.† A successful case of tracheotomy in a boy, æt. 5, who had swallowed a plum-stone, which was expelled during a fit of coughing, through the opening in the trachea.‡ [The conclusion to be drawn from these and other cases alluded to] in the course of the discussions that have taken place, is certainly in favour of the performance of tracheotomy.]

*Lungs, abscess of.* M. Aran§ has published a valuable memoir on abscess of the lungs, under which designation he comprises every collection of pus, in an accidental or abnormal cavity, formed at the expense of the organ, by the separation or destruction of its molecules, from whatever cause it may originate. He, therefore, divides pulmonic abscesses into—1, Phlegmonous; and, 2, Metastatic, or symptomatic; placing in the former category those which succeed to softening of tubercles. He then proceeds to point out the rarity of the true phlegmonous abscess, and refers to most of the recorded cases, distinguishing the forms presented. In describing the anatomical characters he notices the fact that gangrene of the lungs is sometimes only a consequence of abscess, quoting an example from Andral (Clin. Méd. t. ii, p. 299,) and giving an analogous case observed by himself. In fourteen of twenty-four cases the abscess was seated in the right lung [as might be expected, from the greater frequency of pneumonia of the right lung,] in six in the left lung, and in two both lungs were affected. In ten cases the upper lobes were the seat of the abscesses, and in five the lower. [One, among other proofs, that might be adduced to show that pneumonia of the upper lobe is not so rare as has been represented.] The abscesses are generally covered by a layer of pulmonary tissue, and are seldom deeply seated. The following are the ages of 28 patients:—2 under 20; 12 between 20 and 40; 9 from 40 to 60; and 5 from 60 to 70. 11 were women and 17 men. The majority had had their health deteriorated by excess of work or previous sickness; but a certain number were of strong athletic constitution and sanguineous temperament. [The author of this Report once met with a pneumonic abscess in a child between three and four years of age, who died of croup, with which the case was complicated. The abscess occupied the centre of the upper lobe of the left lung. The surrounding lung was everywhere permeable to air, except the portion forming the walls of the abscess and that which intervened between it and the pleuræ separating the two lobes, which were adherent from pleuritic inflammation. No tubercles existed, and the contents of the abscess consisted of pure pus.] *The diagnosis* of pulmonary abscess M. Aran considers impossible, as long as no communication exists with the bronchi or pleura. After this the diagnosis between this lesion and tubercular cavities, or those resulting from pulmonary apoplexy and dilated bronchi, is to be determined —1, by the seat, in the middle and inferior parts of the lung [? see above;] 2, the presence of bronchial respiration and crepitant râle around the excavation; 3, the slow progress of the disease; 4, the continuance of embonpoint and strength; and, lastly, by the previous existence of pneumonia. M. Aran at-

* Provincial Med. and Surg. Journal, Sept. 2, 1843, by Edwin Casson. † Idem. ‡ Idem.
§ Gazette Médicale de Paris, Sept. 24 and Oct. 8, 1842.

tempts to show that cicatrization of pulmonic abscesses is not so rare as represented, by stating that of 59 cases which he has collected, 29, or about half, thus terminated! He adduces one tolerably satisfactory case, occurring under his own care. His observations on the treatment and on the other forms of the disease, offer nothing new or interesting.*

A case of *gangrene of the lung*, terminating favorably, is recorded by Dr. A. Szerlecki,† which, though well marked in so far as respects the existence of gangrene, does not justify the designation given to it of idiopathic gangrene, unpreceded by pneumonia. The favorable result is attributed to the use of acetate of lead and opium. A less satisfactory case, as regards the diagnosis, is given by Dr. Zurkowski,‡ in which the cure is attributed to acetate of lead, creasote, and galbanum. In connexion with this subject, reference may also be made to a curious case detailed by Professor Romig, of Grau,§ in which portions of healthy lung were expectorated. The patient, æt. 34, had for some years been subject to moist cough, unattended by pain. In the course of an anomalous illness, characterized chiefly by abundant hemorrhage from the bowels, cough and dyspnea came on, and during a violent fit of coughing he brought up a portion of apparently healthy pulmonary substance, one inch and a quarter long and half an inch broad and thick, of the colour and appearance of healthy lung, and which swam in water. On the following day another similar but smaller portion was expectorated. Reference is made to a similar case recorded in the second number of the Central Zeitung, by Dr. Joel, of Berlin.

*Pleurisy, Diagnosis.* The existence and characters of bronchial respiration in pleuritic effusions have attracted considerable attention in France. That the sound of respiration is not obliterated in pleurisy has been maintained by M. Hirtz, Andral, Cruveilhier, and many others. M. Monneret has given his experience on this subject.** The sound, he says, in most cases resembles that of expiration as heard under the clavicles in different stages of pulmonary phthisis. Usually the inspiratory sound is scarcely appreciable, and the abnormal sound accompanies expiration only. When both inspiration and expiration are heard, the latter is always the most intense. Though, in many cases, the "souffle" of pleurisy differs from that of pneumonia, it presents various shades, and cannot be distinguished by its "timbre" alone. It is usually heard over the inferior angle of the scapula and its lower third, or even as high as the spine of the scapula, and along its inner border. Wherever the tubular souffle of pleurisy is heard, œgophony (not brochophony) is also present (?) and dulness on percussion extends as high as the spine of the scapula. Five cases are given, corroborating the above statements, and in which the true symptoms and signs of pneumonia were absent, and the treatment such as would not have proved sufficient in pneumonia.

M. Netter†† also states that he has found bronchial respiration to be a frequent phenomenon in pleurisy, and points out the intimate connexion between œgophony and the pleuritic "souffle,' the latter being as constant as the former. In every case in which œgophony was present, the bronchial murmur accompanied expiration, and was sometimes feeble, of short duration, and metallic in its character. The latter circumstance he considers important, as explaining the nature of œgophony. He rejects Laennec's explanation of this phenomenon, which he states he has met with when the fluid effused was considerable. He in fact believes it to be dependent on the bronchial murmur, and affirms that the former is the more trembling, and stuttering, in its character, in proportion as the latter is stronger. Dr. Chambers, of Colchester,‡‡ has found "a gentle gurgling sound," as if produced by the rolling or displacement of a fluid, to be

* See also a case of extensive purulent infiltration and abscess of the right lung, in Provincial Medical and Surgical Journal, Oct. 8, 1842.

† Schmidt's Jahrbücher, No. xi, 2 Heft, 1844.

‡ Ibid. same date.

§ Allg. Med. Centr. Zeitung, Oct. 29, 1842.

|| Archives Gén. de Méd., 2de Ser. t. xiii.

** Gazette Méd. de Paris, Dec. 31, 1842, and Gazette des Hôpitaux, 1 Nov. 1842.

†† Gazette Méd. de Paris, 6 Jan. 1843; Archives Gén. de Méd. March, 1843.

‡‡ Lancet, May 4, 1844.

an invariable attendant of pleuritic effusion. It is most readily detected in the reclining posture. M. Damoiseau* insists on the importance of a friction sound occurring on the absorption of the liquid, as diagnostic of its disappearance, and which sound he states, is preceded by a crepitus resembling that of pneumonia. Dr. Levy, of the Val de Grace, confirms most of M. Netter's statements.†

Dr. Zechmeister,‡ from observations made on a number of cases [how many he does not say] concludes that the decubitus of pleuritic patients is of no diagnostic value, though he states that in the acute stage, when there is much pain and fever, they always lie, either on the sound side, or inclining to the sound side; and in the opposite postures, in the later stages, when there is no pain either on pressure, or from the thoracic movements.

*Empyema, Diagnosis of.* Mr. M'Donnels' 'Contributions to the Diagnosis of Empyema with cases,'§ are exceedingly valuable, and contain new and important views of several points connected with this affection, which claim careful attention. The first part of his paper contains three cases, two original and one reported by Dr. Croly, (Medical Press, vol. 8, p. 135) of 'Pulsating Empyema of Necessity.' These cases, "perfectly new in the history of empyema," presented "large pulsating tumours in the situation usually occupied by the apex of the heart," which organ, in all the cases, "was dislocated to the right of the sternum." The tumours were, in fact, abscesses formed by purulent matter effused from the pleura, and making its exit in the vicinity of the heart. This organ, pushed out of its normal position, pulsated strongly and equally against the walls of the abscesses, the contents of which being fluid and of equal density, communicated a uniform diastolic impulse, without thrill, or bruit de soufflet, to all the surrounding parts, but most intense nearest the source of pulsation. In two of the cases, besides the tumour in the situation of the heart's apex, others existed posteriorly between the tenth and eleventh ribs, which, from their size (that of a hen's egg), situation, and more feeble pulsation, were more likely to lead to the supposition of aneurism. The remarks which Mr. M'D. makes on the co-existence of purulent expectoration, with empyema, are important. In his first case, the patient having been labouring, for some time, under severe diarrhœa, expectorated in the course of one day, as much as a pint of greenish pus, and the diarrhœa was suddenly checked. On examination, post mortem, no trace of communication could be detected between the sac of the empyema and the bronchi, the lining membrane of which was perfectly sound, and free from all signs of inflammation. He therefore considers the case as offering an illustration of vicarious action of the mucous membranes of the lungs and intestines, by which an evacuation of pus is effected, and a corresponding diminution of the empyema occurs. "Purulent expectoration, of frequent occurrence in empyema, is often indicative merely of an effort of nature to get rid of the purulent collection by the readiest outlet." On the condition of the sound lung it is observed, "that though true bronchitis sometimes occurs, when the lung of the affected side is so compressed and bound down by adhesions as to be unable to take any part in the respiratory process;" "not infrequently, mere congestion of the mucous membrane will give rise to all the *physical signs of bronchitis*, or some of the stethoscopic signs of pneumonia, to which too much importance should not be attached." It has been generally supposed that the only way in which the *liver* is engaged in empyema, is by being depressed mechanically, when extensive effusion of the right side exists; and its condition in empyema of the left side has been overlooked. Mr. M'D. takes a no less new, than ingenious view of this subject. Epigastric and hypochondriac tumours occur in empyema of the left side, as well as of the right; and in both cases are to be ascribed not so much to mechanical displacement (which is not denied,) as to actual enlargement of the liver, from congestion, "*analo-*

* Archives Gén. de Méd. Oct. 1843.

† Gazette Méd. de Paris, 6 Jan. 1843.

§ Dublin Journal of Med. Science, March, 1844.

*gous to what takes place in morbus cordis, and diseases of the lungs, attended with imperfect æration of the blood.*" [The accounts given of the state of the liver by many authors (who have not taken this view of the subject) are confirmatory of Mr. M'D.'s views, which are certainly consonant with established laws.]

Dr. Krause,* who has supplied a good summary on the whole subject of empyema, and many valuable tables, illustrating various statistical points, though he remarks that disease of the liver is but a rare attendant on empyema, by his statements with reference to that organ, where its condition is mentioned, certainly confirms Mr. M'D.'s views. Some curious instances are given by Dr. Krause, of the modes in which the purulent collections are sometimes discharged. In one case, the matter made its exit through a small opening in the diaphragm, passed along the side of the lumbar vertebræ, and was discharged under the form of psoas abscess, beneath Poupart's ligament. In another, a communication was established between the intestines and the chest; and fæcal matter introduced into the thorax. In reference to the artificial discharge of pleuritic effusions, and the circumstances calling for the performance of paracentesis thoracis opinions continue to be much divided. Dr. Krause is decidedly against having recourse to the operation at an early period, and considers it much more likely to be useful when the tendency to inflammation is abated.† Dr. Hamilton Roe‡ states, that on a review of thirty-nine cases recorded in the British journals between the years 1812 and 1842, he found that only eleven had died. Twenty-four cases fell under his own observation, the results of which led him to conclude that the operation is as free from danger as any other performed on the human body, and that it is usually successful when employed early, either in empyema or inflammatory hydrothorax: the common cause of failure being the late period at which it has been performed. His experience has led him to associate bulging of the intercostals with purulent effusion, and non-bulging with the effusion of serum. Of twenty cases, on which the observations of Dr. Hughes and Mr. Cock§ are founded, seven were completely cured; three recovered partially; nine died (six of whom were phthisical); in one the fluid was not reached by the trochar; another was sinking at the time of the operation; and in the remaining case, death occurred suddenly, (there being hydrothorax of the opposite side); one case was still under treatment. In no instance could the fatal event be said to have been hastened by the operation. In doubtful cases the use of Dr. Babington's exploratory trochar is recommended. Dr. Theophilus Thompson records a successful case after repeated punctures, in a boy æt. 5;|| and Dr. Gadechens in a boy æt. 3.** Two cases are related in which electro-puncture was tried; in one a very marked and rapid absorption of the fluid took place after two or three applications of the remedy.††

*Pneumothorax.* Dr. Hughes's essay on this subject‡‡ contains a brief general history of the affection, founded on already published cases, especially those which have occurred in Guy's Hospital, with critical remarks on the more important phenomena. His principal conclusions are, that pneumothorax has not been proved to arise from other causes than a communication of the pleura with the external air;—that it may occur as a consequence of phthisis with a very small, or without any cavity in the lung;—that it may take place without the occurrence of any symptoms by which the period of the accident can be fixed;—that the greater the amount of disease in the lung, and the more extensive the adhesions of the affected side, the less marked and characteristic are the indications of the disease;—that it is not insusceptible of cure, and in some cases, of advanced phthisis, may prolong life. A very interesting case is recorded by Dr. Barker,§§ occurring in a man convalescent

* Das Empyem u. seine Heilung, von Dr. A. Krause, 8vo; Danzig, 1844.
† Op. cit.
‡ Medical Gazette, May 3, 1844.
§ Guy's Hospital Reports, 2d Ser. No. iii.
|| Medical Gazette, May 3, 1844.
** Oppenheim's Zeitschrift, Dec. 1843, p. 540.
†† Gazette des Hôpitaux, 25 Mars, 1843.
‡‡ London Med. Gazette, vol. i, 1843-4, p. 434, &c.
§§ Medical Gazette, Nov. 10, 1843.

from fever, in St. Thomas's Hospital, and in whom all the ordinary symptoms were, for a long time, masked, owing to extensive pleuritic adhesions. Fluid was found effused into the lower portion of the pleura, and a small gangrenous spot on the lower lobe of the lung from which an eschar had fallen; both lungs were otherwise healthy.

*Phthisis.* On the Statistics of Phthisis in the United States, Dr. Hayward's essay* may be referred to, as containing the results of an investigation into the mortality from that disease, in the cities of Boston, New York, and Philadelphia, during a period of thirty years. The most striking fact shown by his tables, is the great *decrease* of deaths by consumption in those cities, especially in Boston. With a view to determine the comparative frequency and the peculiarities of phthisis in warm climates, M. Rufz† has contributed the results of his observations on the disease, at Martinique, where it appears that with the exception of phthisis, pulmonary complaints are exceedingly rare. He met with but three cases of pneumonia in five years; and chronic bronchitis even among the old is very uncommon. Of 1954 patients out of a population of 17,000, seen in the course of five years, 123 were tubercular, or 13 per cent. In those dying of phthisis, tuberculization of other organs besides the lungs is much less frequent than with us: diarrhœa very rare. Other allied scrofulous diseases are uncommon. Mr. Wells‡ remarks on the injurious influence on the phthisical patients of Malta, exerted by the scirrocco and liebeccio winds from the shores of Syria and Lybia. The depressing influence of these warm winds was great on all pulmonary complaints, and incipient cases of phthisis ran rapidly into a confirmed and incurable state. The importance of chamber warmth and protecting raiment as counteracting the exciting causes of phthisis, has been insisted on by Sir George Lefevre§ as the result of his observations of the rarity of pulmonary disease in Russia. In reference to the *diagnosis* of phthisis, Dr. Hughes|| has afforded some important statistical information on its location. From the records of 250 cases, it appears that the left lung was chiefly diseased in 116, and the right in 89. The upper lobe of one or both lungs, was solely or principally diseased in 237, or 95 per cent. With these results, a paper by the same author, on the location of pneumonia,** may be usefully compared. Dr. Hamernjk†† remarks that the respiration is sometimes natural in the sub-clavicular region, when there is diminished resonance. There is no difficulty in the diagnosis in such cases; but it sometimes happens that the respiratory and bronchial sounds of other diseased parts are transmitted even to the above regions, when we may be led into error. He gives a case in which all the phenomena of phthisis were present, but contradicted by the autopsy. Among other signs there were deficiency of respiration in the sub-clavicular region, and dulness on percussion. On dissection chronic catarrh and emphysema were detected, but no tubercles. Alluding to the diagnostic value of the expiratory phenomena, Mr. Wells states‡‡ that in the earliest stages, before any dulness or any general symptoms, led to the suspicion of phthisis, alteration in the intensity of the expiratory murmur excited fears of the existence of tubercles, which invariably proved well founded.

*Treatment of phthisis.* To say that nothing new or important has been advanced on the treatment of phthisis, amidst all the marvels that have been announced, some persons may think strange. Very little, however, has unfortunately appeared that can be made available for this Report. M. Max, Simon§§ opposes the notion that phthisis is a chronic pneumonia, and offers his own testimony as coinciding with that of Hufeland, to the curability of phthisis. Among other remedial means he distinguishes the ferruginous pre-

* New England Quarterly Journal, Jan. 1843. See also on this subject, Boston Med. Journal, March 1, 1842; and Rev. Médicale, Jan. Feb. and March, 1842, par M. Briquet.

† Mém. de l'Acad. Roy. de Méd. t. x.

‡ Edinb. Med. and Surgical Journal, April 1844.

§ Lancet, vol. i, 1842-3, p. 197.

|| Guy's Hospital Reports, vol. vii.

** Ibid.

†† Allg. Med. Centr. Zeitung, Nov. 9, 1842.

‡‡ Loc. cit. Report of Malta Hospital.

§§ Bulletin Gén. de Thérap. 15 et 30 April, 1843.

parations as the most powerful. He insists on their power of rendering a healthy state of organism to pallid, lymphatic persons; and in support of this quotes some of the experiments of Andral and Gavarret. He has commenced some experiments to ascertain the effects of these medicines on children during their first year, by giving iron to the mothers, and states that some pale anæmic feeble children have thus been visibly strengthened. He hints at the possibility of thus removing the congenital predisposition to tubercular disease. M. Sandras,* in giving in his report on the Memoir of M. Pereyra of Bordeaux, on the diagnosis and treatment of phthisis,† refutes all his statements, and says that he had tried the cod-liver oil on thirty patients, many of whom took it with difficulty. In no single instance was any benefit derived. He attributes all the good which it may effect in scrofula to the iodine it contains, and which, he very justly says, it is better to give in a more simple form. [For various statements allied to those of M. Pereyra, on the treatment of phthisis by naphtha, paracentesis, &c., the reader is referred to previous numbers of this Journal, where these statements have already received sufficient notice.] Dr. Durrant‡ has called attention "to the large amount of benefit which, in a great majority of instances is to be derived from a persevering use of emetics" in the incipient stages of phthisis; a mode of treatment which has already received the sanction of more than one high authority. On the curability of tubercles in the lungs and bronchial glands, some startling, not to say incredible, statements have been made by M. Boudet to the Academy of Sciences.§ Having examined a great number of bodies without regard to the disease or the age of the patients, he finds that from one to two years of age, tubercles in the lungs or bronchial glands occur once out of 57; from two to fifteen years, in 3 out of 4; and from fifteen to seventy-six years, in 6 out of 7. In adults, therefore, the presence of tubercles is the rule, and their absence the exception! This extraordinary statement is explained by the following considerations: That in many instances, the tubercles are few, limited, and in a great number of cases, so transformed that they exert no injurious influence on the health. A favorable termination is rare in infancy; before three years of age he has met with only one example; but from three to fifteen years of age he has seen 12 instances out of 45 cases. From fifteen to seventy-six years of age he has found tubercles cured, either in the lungs or bronchial glands, in 97 out of 116 cases! In 61 of these 97 cases, "the fortunate termination appeared definite, and the rest of the organ contained no recent tubercle." He then proceeds to point out the various modes in which these particular results are brought about, the whole merit of which is ascribed to nature.

*Phthisis from the inhalation of gritty particles.* Dr. Holland has published the results of his observations on the peculiar form of pulmonary disease to which the grinders of Sheffield are liable, and by them commonly called grinders' asthma or rot. The disease is shown to arise from the inhalation of metallic and other gritty particles, the noxious influence of which is first exerted on the mucous membrane of the trachea and bronchi, and subsequently on the lungs. The symptoms, up to an advanced period of the affection, differ materially from those of tubercular phthisis, there being much less evidence of constitutional disturbance. Quickness of pulse, impaired digestive powers, diarrhœa, emaciation, and hectic being far less prominent symptoms, and for the most part not occurring till the very close of the disease. The local symptoms, in one class of cases, are rather those of chronic bronchitis and asthma than of ordinary phthisis, cough being for years the principal symptom. In another class the contraction and flattening of the chest, and dulness on percussion, present more of the characters of tubercular disease of the lungs, with which also the constitutional symptoms in this class more accord. In both sets of cases, according to Dr. Holland, tubercles are found in the

* Revue Méd. March 1844, p. 450.
† Vide No. 37 of this Journal, p. 140.
‡ Medical Gazette, May and June 1843.
§ Gazette des Hôpitaux, Jan. 19, 1843.

lungs, though by no means invariably in the first class of cases. [But the structural changes are very imperfectly described, and no sufficient evidence is given of the existence of true tubercles.] On this point, as well as on some others, Mr. Waterhouse's* account is more satisfactory. He speaks of finding "depositions of foreign matter in the air-cells, and the formation of purulent foci, and collections *resembling* tubercle." The affection is often, he says, connected with the scrofulous diathesis, and then partakes more completely of the character of phthisis. A brief account of the morbid appearances in this disease will be found in Mr. Law's Report of the Sheffield Med. Society, from which it appears that the lungs are much condensed and indurated, and studded (chiefly, and often exclusively, in the upper lobes) with *dark currant-like bodies*.†

Dr. Petrenz‡ has described the progress and nature of the pulmonary disease to which the stone quarriers of Schaudun are liable, which is clearly analogous to that occurring to the Sheffield grinders. There is the same absence, for a long time, of the constitutional symptoms characteristic of phthisis. Masses of gritty solid matter are often mixed with the expectoration. The same recklessness of life and manners which mark the grinders characterize these stone-cutters, and the cases are often modified accordingly. The disease is not hereditary. Most of the workmen die under 40. The account of the post-mortem appearances shows the marked distinction between the pulmonary destruction induced by mechanical irritation and chronic inflammation, and that which arises from the deposition of tubercle.

*Pneumonia in the old, treated without bloodletting.* Dr. V. Röderer§ remarks on the high mortality (one half to two thirds) attending the cases of pneumonia, occurring in the aged, when treated in the usual way by bloodletting, even when employed with activity from the first onset of the disease. Dr. R. has, therefore, been led to abandon bloodletting (to which he thinks the mortality in great measure attributable,) and employ tartar emetic in conjunction with opium. In the first and second stages of primitive simple pneumonia in the old, he gives from 4 to 6 grs. of tartar emetic with an equal quantity of opium, in the course of twenty-four hours, either in the form of pill or in solution. He was seldom obliged to continue this plan longer than 4 or 5 days. He abstained from all bleeding and blisters, confining himself to the above treatment, except in some rare cases where aperient enemata were required. Of forty-two patients thus treated he lost only thirteen; these were cases where the diagnosis was made with all accuracy, and the progress carefully marked. A lengthy memoir, by Mr. E. M. Martin,|| founded on sixty-seven cases, occurring during the winter of 1843, in the wards of M. Prus, at Salpêtrière, is intended to point out the chief differences between pneumonia in the adult and the aged. The apex of the lung was much more frequently the seat of the inflammation than in the adult. It was either very general or of very limited extent. Abscess was much more frequent, occurring in 3 cases out of 67; (in 5 of 70 cases detailed by Mercier at Bicêtre abscesses were found;) emphysema very frequently co-exists. The mode of invasion was sometimes gradual and sometimes sudden; but all the symptoms which attracted the patients' attention were referrible to the stomach, e. g. bilious vomiting.**

*Bronchial glands.* Dr. Golding Bird†† has detailed the particulars of a case of phthisis, complicated with scrofulous disease of the cervical and bronchial glands. The patient, a girl æt. 16, presented physical indications of tubercular disease of the right lung, while, on the left side the respiration gradually became more and more feeble, without any loss of sonoriety, but with corresponding dyspnœa, and indications of cerebral congestion. Under these symptoms she sank, without emaciation, cough, expectoration, or any of those signs

* Provincial Medical and Surgical Journal, Sept. 16, 1843. † Ibid. June 12, 1844.

‡ Hufeland's Journal, April 1844, in Schmidt's Jahrbucher. § Oester. Med. Wochensch. Jan. 1843.

|| Révue Méd. Jan. 1844.

** On the Mortality of Pneumonia as influenced by age, sex, seat, &c.; see a Paper by Professor Henderson, in Edinburgh London Monthly Journal. †† Medical Gazette, Nov. 11, 1842.

which mark the close of ordinary cases of phthisis. From the post-mortem examination it appeared that the immediate cause of death was constriction of the left bronchus by enlarged bronchial glands, so as to prevent the ingress and egress of air to the left lung, the right being full of tubercles. Dr. Bird has met with two other similar cases. In a case related before the Med. Chir. Soc. by Dr. Graham Tice,* the principal symptoms were dyspnea, cough, foul taste in the mouth, and subsequently stridulous breathing. Vesicular respiration disappeared on the right side completely, and partially on the left, whilst the chest sounded well on percussion. Death took place suddenly. The lungs were found healthy. The bifurcation of the trachea was surrounded by a mass of enlarged and suppurating bronchial glands, from which had been discharged a calcareous mass, that was partially engaged in the right bronchus, wholly obstructing its canal.

*Carcinoma of the lung.* Several cases of this disease have been recorded. One by MM. Lionet and Legrand,† in an old man æt. 62 The symptoms were extreme emaciation, dysphagia, percussion clear, respiration feeble, scarcely audible; no abnormal sound attending either inspiration or expiration; neither cough, hemoptysis, nor oppression; death from asthenia. The lungs, like the body, generally were atrophied, and on the summit of each was a cancerous mass, attaching the lungs to the chest by strong adhesions. The case had been mistaken for gastritis, owing to the irritability of stomach. A very similar case is detailed by Dr. Maclachlan‡ but in this instance there was eventually complete dulness of the whole right side, which appeared to move "en masse." The patient was a man æt. 62, and the disease involved the whole right lung. The diagnosis in this case was not made out. The case related by Mr. W. Clark§ was that of a man æt. 45. The symptoms at first were neuralgic pains of the chest, succeeded by cough, dyspnea, rapid pulse, hemoptysis, diminished respiratory murmur, and dulness on percussion: subsequently enlargement, distension, and immobility of the right side, displacement of the liver, and of the heart rather to the left, general venous congestion, and anasarca. The patient died, suffocated, in five or six months from the first attack of neuralgia. The lung was compressed at the posterior part of the chest by an enormous cancerous mass, occupying the whole pleura. Dr. Burrows's case‖ occurred in a married woman, æt. 20. The first symptoms were pain beneath the sternum, cough, expectoration, dyspnea, and some swelling of the cervical glands. Subsequently hemoptysis and currant-juice sputa, paroxysmal cough, enlarged cervical glands and veins on the right side, and weak, husky voice. The physical examination indicated both consolidation of the lung and pleuritic effusion. Four pints of brown fluid were found in the pleura, and white, solid, suet-like masses, containing a creamy fluid, occupied the lower and middle lobes of the lung, and the right bronchus and pulmonary vein contained carcinomatous matter.

Mr. Arrowsmith** has detailed a case of *pulmonary hydatids* the chief symptoms of which were cough of a paroxysmal character, no dyspnea, except after the fits of coughing; dulness over the left upper part of the thorax, then bloody sputa, dyspnea, night perspiration, and diarrhœa. The man ultimately recovered, having at different times expectorated a good many hydatids.

* Lancet, vol. i, 1142-3.

† Gazette des Hôpitaux.

‡ Lond. Med. Gazette, March 31, 1843.

§ Med. Gazette, April 21, 1843.

‖ London Med. Gazette, p. 696, vol. i, 1843-4.

** Provincial Medical and Surgical Journal, Jan. 6, 1844.

†† Archives Gén. de Méd. 1844, p. 466.

